# Deciphering the Contribution of Oriens-Lacunosum/Moleculare (OLM) Cells to Intrinsic θ Rhythms Using Biophysical Local Field Potential (LFP) Models

**DOI:** 10.1523/ENEURO.0146-18.2018

**Published:** 2018-09-04

**Authors:** Alexandra P. Chatzikalymniou, Frances K. Skinner

**Affiliations:** 1 Krembil Research Institute, University Health Network, Toronto, Ontario M5T 058, Canada; 2 Department of Physiology, University of Toronto, Toronto, Ontario M5S 1A8, Canada; 3 Departments of Medicine (Neurology) and Physiology, University of Toronto, Toronto, Ontario M5S 1A8, Canada

**Keywords:** computational modeling, hippocampus, interneurons, local field potential, microcircuit, θ rhythms

## Abstract

Oscillations in local field potentials (LFPs) are prevalent and contribute to brain function. An understanding of the cellular correlates and pathways affecting LFPs is needed, but many overlapping pathways *in vivo* make this difficult to achieve. A prevalent LFP rhythm in the hippocampus associated with memory processing and spatial navigation is the θ (3–12 Hz) oscillation. θ rhythms emerge intrinsically in an *in vitro* whole hippocampus preparation and this reduced preparation makes it possible to assess the contribution of different cell types to LFP generation. We focus on oriens-lacunosum/moleculare (OLM) cells as a major class of interneurons in the hippocampus. OLM cells can influence pyramidal (PYR) cells through two distinct pathways: by direct inhibition of PYR cell distal dendrites, and by indirect disinhibition of PYR cell proximal dendrites. We use previous inhibitory network models and build biophysical LFP models using volume conductor theory. We examine the effect of OLM cells to ongoing intrinsic LFP θ rhythms by directly comparing our model LFP features with experiment. We find that OLM cell inputs regulate the robustness of LFP responses without affecting their average power and that this robust response depends on coactivation of distal inhibition and basal excitation. We use our models to estimate the spatial extent of the region generating LFP θ rhythms, leading us to predict that about 22,000 PYR cells participate in intrinsic θ generation. Besides obtaining an understanding of OLM cell contributions to intrinsic LFP θ rhythms, our work can help decipher cellular correlates of *in vivo* LFPs.

## Significance Statement

Oscillatory local field potentials (LFPs) are extracellularly recorded signals that are widely used to interpret information processing in the brain. θ (3–12 Hz) LFP rhythms are correlated with memory processing, and inhibitory cell subtypes contribute in particular ways to θ. While a precise biophysical modeling scheme linking cellular activity to LFP signals has been established, it is difficult to assess cellular contributions *in vivo* to LFPs because of spatiotemporally overlapping pathways that prevent the unambiguous separation of signals. Using an *in vitro* preparation that exhibits θ rhythms and where there is much less overlap, we build biophysical LFP models and uncover distinct inhibitory cellular contributions. This work brings us closer to obtaining cellular correlates of LFPs and brain function.

## Introduction

Oscillatory brain activities, as can be observed in EEGs and local field potentials (LFPs), are a ubiquitous feature of brain recordings (Buzsáki and Draguhn, 2004). Accumulating evidence indicates that they form part of the neural code by phasically organizing information in brain circuits (Wilson et al., 2015). The LFP is the low-frequency part (<500 Hz) of the extracellular signal. Due to its relative ease of recording, it is commonly used to measure neural activity. It originates from transmembrane currents passing through cellular membranes in the vicinity of a recording electrode tip (Einevoll et al., 2013), and its biophysical origin is understood in the framework of volume conductor theory (Nicholson and Freeman, 1975). Many sources contribute to the LFP (Buzsáki et al., 2012) and depend on the frequency range of the extracellular signal. Slower oscillations (<50 Hz) are generated by synaptic currents as opposed to higher frequency oscillations (>90 Hz) which are influenced by phase-modulated spiking activity (Schomburg et al., 2012). Determining the sources of LFP output is highly challenging in general, and contributions from remote and local activities can be non-intuitive (Herreras, 2016; Carmichael et al., 2017). In essence, it is far from clear how to interpret LFP recordings in light of contributions from many different cell types and pathways.

The hippocampus exhibits many LFP activities including θ and γ rhythms (Buzsáki, 2006; Colgin, 2016). In particular, the prominent θ rhythm (3–12 Hz) is correlated with spatial navigation and episodic memory, rapid eye movement sleep and voluntary behaviors (Buzsáki, 2002). Recently, direct behavioral relevance of θ LFP rhythm phase-coding was demonstrated by delivering perturbations during specific phases of the θ rhythm to preferentially affect encoding or retrieval behaviors (Siegle and Wilson, 2014). This was done by optogenetically stimulating particular inhibitory cell types in the dorsal CA1 region of the hippocampus. Such exciting studies and several reviews (Klausberger and Somogyi, 2008; Kepecs and Fishell, 2014; Hattori et al., 2017) make it clear that the specifics of inhibitory cell types are fundamental to neural coding and brain function. In essence, if we are to understand the brain’s code, i.e., behavior-related changes in oscillatory activity, we need to understand how various cell-type populations contribute to LFP recordings.

A whole hippocampus *in vitro* preparation has been developed and spontaneously generates intrinsic θ (3–12 Hz) rhythms (Goutagny et al., 2009). Given the combination of its reduced nature and robust rhythms, this preparation presents an opportunity to understand cellular contributions to LFP θ rhythms as we can remove several complicating factors by not needing to consider various pathways that exist in *in vivo* scenarios. Ambiguities are greatly reduced and our ability to understand cellular contributions to LFP recordings is greatly enhanced. Oriens-lacunosum/moleculare (OLM) cells are a major class of GABAergic interneurons (Maccaferri, 2005). They play an important role in gating information flow in the hippocampus by facilitating intrahippocampal transmission from CA3 while reducing the influence of entorhinal cortical inputs (Leão et al., 2012). Since OLM cells project to the distal dendrites of pyramidal (PYR) cells they would be expected to generate large LFP deflections due to larger dipole moments (Pettersen et al., 2012). However, these expectations may need to be modified since in addition to inhibiting distal layers they can have an effect on inner and middle layers, since they inhibit interneurons that target PYR cells at those layers (Leão et al., 2012).

In this article, we use computational modeling to determine the contribution of OLM cells to ongoing intrinsic LFP θ rhythms considering their interactions with local targets using the *in vitro* whole hippocampus preparation context. We take advantage of a previous modeling framework of inhibitory networks (Ferguson et al., 2015) and generate biophysical LFP computational models, and investigate the factors that influence θ LFP characteristics. By directly comparing our LFP models with experiment, we are able to constrain the required connectivity profile between OLM cells and other inhibitory cells types, as well as to show that OLM cells control the robustness, but not the power, of intrinsic LFP θ rhythms. We are also able to assess the spatial reach of the extracellular signal and so estimate the number of cells that contribute to the LFP signal. In general, we show how the many complex interactions lead to emergent LFP output that are non-intuitive and would not be possible to understand without biophysical LFP modeling in an experimentally constrained microcircuit context. As such, our work shows a way forward to obtain an understanding of cellular contributions to brain rhythms.

## Materials and Methods

### Network model details

This work builds on previously developed models described in Ferguson et al. (2015). Here, we provide a summary of specifics that are salient to the present study.

#### Inhibitory cell types and numbers, PYR cell model

**Figure 1.**
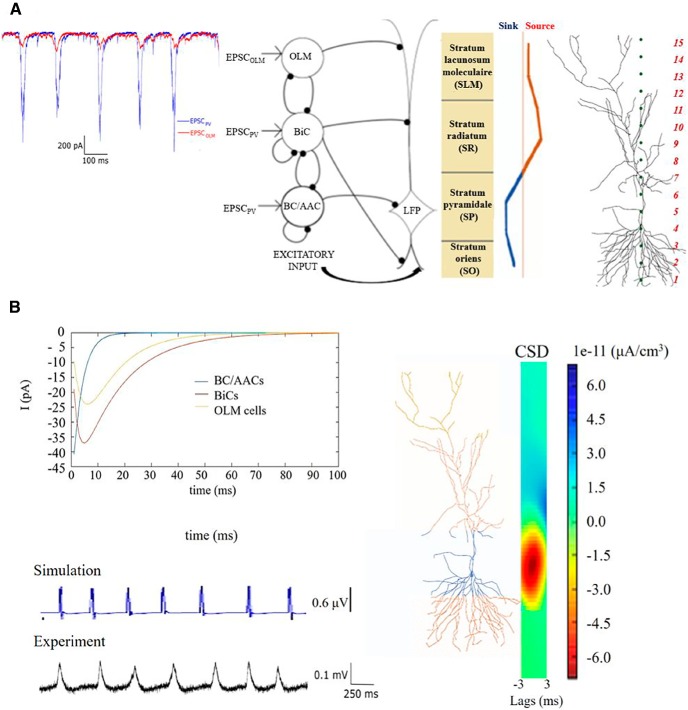
Model setup and experimental essence. ***A***, A schematic of the network model used by Ferguson et al. (2015) is shown in the middle. The network model contains single compartment representations for OLM cells, BiCs, and BC/AACs. Inhibitory synapses are represented by filled black circles. Each inhibitory cell receives EPSCs that is taken from experimental intracellular recordings as shown on the far left (adapted from Ferguson et al., 2015). Each inhibitory cell synapses onto a PYR cell model as schematized. There are 350 OLM cells, 120 BiCs, and 380 BC/AACs. Basal excitatory input is also included. An illustration of the polarity changes (source/sink) seen in the different labeled layers from LFP experimental recordings is shown on the right, and the detailed PYR cell morphology that is used along with the 15 equidistant electrode locations in the different layers is shown as red numbers on the far right. ***B***, IPSCs from the different cell types (colored as indicated) are shown on the left to show their different kinetics. Parameter values are given in Table 1, and the same coloring is used on the detailed PYR cell morphology to indicate the synaptic location regions for the different cell types. An example simulation of a computed LFP from the SR layer (using parameter values of *g_sb_* = 6 and *g_bs_* = 1.25 nS, *c_sb_* = 0.21) is shown below, and the computed CSD is shown on the right (averaged over time). On the bottom is an example of an experimental LFP recording from the SR layer (adapted from Ferguson et al., 2015).

The inhibitory network model consists of 850 cells and represents a volume of 1 mm^3^ as shown to be appropriate to obtain spontaneous θ rhythms in the *in vitro* whole hippocampus preparation (Ferguson et al., 2013, 2015; Goutagny et al., 2009). Four different types of inhibitory cells are included: basket/axo-axonic cells (BC/AACs), bistratified cells (BiCs), and OLM cells. BC/AACs comprise a 380-cell population and target somatic, perisomatic, and axo-axonic regions of PYR cells. The BiCs comprise a 120-cell population and target middle, apical and basal regions of PYR cells, and the OLM cells comprise a 350-cell population and target the distal, apical dendrites of PYR cells. As in Ferguson et al. (2015), the structure of the PYR cell model was based on the one used in Migliore and Migliore (2012) as implemented in the NEURON simulator (Carnevale and Hines, 2006; see ModelDB accession number 144541). The PYR cell model was used as a passive integrator of inputs from cell firings at the various layers of the hippocampus, and all active, voltage-gated channel conductances were set to zero. This overall network model is schematized in Figure 1*A*. With the exception of basal excitatory input, it is the same as used in Ferguson et al. (2015).

#### Inhibitory cell models and drives

The inhibitory cell models are single compartment, have an Izhikevich mathematical structure (Izhikevich, 2003) and were constructed by fitting to experimental data from whole-cell patch clamp recordings in the whole hippocampus preparation (Ferguson et al., 2015). All of the cell model parameter values are given in Ferguson et al. (2015). Parvalbumin-expressing (PV) cell types are BC/AACs and BiCs, and somatostatin-expressing (SOM) cell types are OLM cells. Each cell model is driven by excitatory postsynaptic currents (EPSCs) taken directly from experiment (Huh et al., 2016) during ongoing spontaneous θ rhythms for PV or SOM cells. The EPSCs were designed to ensure that the inhibitory cells receive frequency-matched current inputs and at the same time have amplitudes and peak alignments that were consistent with θ oscillations in experiment (Ferguson et al., 2015; see EPSC*_PV_* and EPSC*_OLM_* examples in Fig. 1*A*). Importantly, the experimental variability in amplitude and timing of EPSCs across cells was captured by varying the gain (factor by which the EPSC was scaled to alter the amplitude) and timing of the EPSCs across cells with a normal distribution in accordance with the experimental recordings. Thus, each inhibitory cell model received a unique set of excitatory synaptic inputs reflecting the range of amplitudes and timing of those recorded experimentally.

#### Inhibitory network connectivity and output

PV cells (BC/AACs and BiCs) were randomly connected with probabilities and synaptic conductance values based on experimental estimates from the literature and previous modeling work (Ferguson et al., 2013). Connections between BiCs and OLM cells are known to exist (Leão et al., 2012) and a range of values from the literature was previously estimated, with the connection probability from BiCs to OLM cells taken as 0.64 times the connection probability from OLM cells to BiCs (Ferguson et al., 2015). Although OLM-BiC connections exist, their synaptic conductance values are unclear but can be roughly estimated from the literature. In previous work, the balance of parameter values important for θ rhythms was specifically examined by exploring a wide range of values that encompassed determined estimates (Ferguson et al., 2015). Inhibitory synapses were modeled using a first order kinetic process with appropriate rise and decay time constants. The spiking output of the inhibitory network models briefly described here were computed for the range of synaptic conductance strengths and connection probabilities given in Table 1. For the work in this paper we used output from these inhibitory networks. Specifically, these simulations were done for 5 s; the connection probability from OLM cells to BiCs (*c_sb_*) varied from 0.01 to 0.33 with a step size of 0.02 producing 16 sets of connection probabilities; synaptic conductance values ranged from 0 to 6 nS for OLM cells to BiCs (*g_sb_*) and for BiCs to OLM cells (*g_bs_*). By changing *g_sb_* and *g_bs_* with a step size resolution of 0.25 nS, 625 raster plots were produced. So the total number of raster plots in our study here as computed in Ferguson et al. (2015) is (625 × 16) 10,000, and they are all available on Open Science Framework (osf.io/vw3jh).

**Table 1.** Connectivity parameter values

Cell type X to cell type Y (X – Y)	Connection probability	Maximal synaptic Conduc.(nS) or synaptic weight (μS) to PYR cell	Synaptic rise time (ms)	Synaptic decay time (ms)
BC/AAC – BC/AAC	0.12	3	0.27	1.7
BC/AAC – BiC	0.12	3	0.27	1.7
BC/AAC – OLM cell	0	N/A	N/A	N/A
BC/AAC – PYR cell	1	0.00038	0.3	3.5
BiC – BC/AAC	0.12	3	0.27	1.7
BiC – BiC	0.12	3	0.27	1.7
BiC – OLM cell	0–0.224	0–6	2	16.1
BiC – PYR cell	1	0.00044	2	16.1
OLM cell – BC/AAC	0	N/A	N/A	N/A
OLM cell – BiC	0–0.33	0-6	2	16.1
OLM cell – OLM cell	0	N/A	N/A	N/A
OLM cell – PYR cell	1	0.00067	3.5	11.8
Excitatory input	1	0.00044	0.5	3
to PYR cell				
(197 contacts to basal tree)				

N/A = not applicable.

#### Synaptic weights and distribution onto PYR cell

Inhibitory inputs to the PYR cell model were distributed in the same way as done in Ferguson et al. (2015). That is, we distinguished between synapses at the distal layer (stratum lacunosum-moleculare), medial and basal layers (stratum radiatum and oriens), and the perisomatic/somatic layer [stratum pyramidale (SP)]. Distal synapses were defined as those that are >475 μm from the soma; apical and basal synapses were defined as those that are >50–375 μm from the soma; perisomatic/somatic synapses were defined as those that are <30 μm from the soma. We created three lists of components (where each component points to a specific segment of a section in the PYR cell model), for the possible distal, proximal apical/basal, and perisomatic/somatic synaptic targets. For each individual, presynaptic inhibitory cell model, we randomly chose a synaptic location on the passive CA1 PYR cell model from the respective list (distal dendrites for OLM cell models, apical/basal dendrites for BiC models, and perisomatic/somatic locations for BC/AACs). Then the spike times from the individual, inhibitory cell models filled a vector, and an artificial spiking cell was defined to generate spike events at the times stored in that vector at the specific location at which that cell created a synaptic target. We used the Exp2Syn function in NEURON to define the synaptic kinetic scheme of the synapse. This function defines a synapse as a synaptic event with exponential rise and decay, that is triggered by presynaptic spikes, and has a specific weight that determines its synaptic strength, and an inhibitory reversal potential of -85 mV, as measured in the whole hippocampus preparation. Synaptic weight values onto the PYR cell from the different cell populations were estimated using somatic inhibitory postsynaptic current (IPSC) values for OLM cells onto PYR cells (Maccaferri et al., 2000). As these synaptic weights were not clearly known, we used different synaptic weight profiles in the explorations as was been done previously (Ferguson et al., 2015). The main profile used was graded such that the different cell types led to similar somatic IPSC amplitudes, considering that 0.00067 μS can be estimated from the OLM cells IPSCs (Table 1). Several other synaptic weight profiles were examined. Finally, we note that an *ad hoc* representation for LFPs was previously used (Ferguson et al., 2015) as given by an inverted summation of all integrated inputs as measured at the PYR cell soma. That is, the postsynaptic potentials on the PYR cell were due to the various inhibitory cell firings that comprised the presynaptic spike populations.

### Additional network model details for this study

For the study here, inhibitory inputs were distributed in the same way as in Ferguson et al. (2015). In Ferguson et al. (2015), the literature was used to estimate synaptic conductances between OLM cells and BiCs as 3–4 nS, and Bezaire et al. (2016) used 10 synapses/connection as estimates in their detailed data-driven computational models. This implies that a single synapse would be 0.3–0.4 nS, representing an approximate minimum connection weight.

As we made direct comparisons with θ LFP experimental recordings here, it was important to include excitatory input to the PYR cell model. Thus, we also included excitation due to CA1 recurrent collaterals which synapse on basal dendrites (Takács et al., 2012). In Ferguson et al. (2015), excitatory feedback was not included in a direct fashion as the focus was on ongoing θ rhythms and OLM-BiC interactions, and not on θ generation mechanisms explicitly. Thus, model excitatory cell populations were not specifically modeled. This means that we did not have explicit spike rasters for excitatory populations as we did for the inhibitory cell populations. Rather than generate an arbitrary set of spike times to simulate excitatory inputs, we used spike times from a BiC raster (*g_sb_* = 3.75, *g_bs_* = 1.75 nS, *c_sb_* = 0.21), in which the neuron order was randomized, and with comparable synaptic weights. Using these random spike trains we generated spike vectors exactly as in the case of interneurons and randomly distributed them on basal dendrites using 197 synapses based on number estimates from Bezaire and Soltesz (2013) and Bezaire et al. (2016). In this way, we did not have a spatiotemporal dominance of inhibitory or excitatory input in basal dendrites. We used an excitatory reversal potential of -15 mV as measured in the whole hippocampus preparation, and synaptic time constants in line with modeling work (Ferguson et al., 2017). In essence, we simulated EPSCs using random spike trains of θ frequency instead of explicitly modeling PYR cell spiking activity. We note that with these choices, somatically recorded currents in our PYR cell models were similar to what is observed in experiments (Huh et al., 2016). All parameter values are summarized in Table 1.

We note that the inhibitory cell spike rasters computed in Ferguson et al. (2015) used random connectivities between the different inhibitory cell populations. Consider that a given set of parameters (*c_sb_*, *g_sb_*, *g_bs_*) defines a connectivity map. Each cell within a given population is randomly assigned a synaptic location within the boundaries of the dendritic tree on which it projects. Based on a given connectivity map the spiking activity of the various cell populations will differ. Therefore, the characteristics of the produced biophysical LFP will depend on the spike distribution of a given population defined by the connectivity map and also the number and location of synapses on the dendritic tree. To ensure that our LFP output was not dependent on the specific synaptic location that every cell was assigned to, we generalized our observations by performing many trials for a given connectivity map, assigning randomly different location to the cells of each population to ensure that the LFP output was not dependent on that aspect.

### Biophysical computation of LFP

Extracellular potentials are generated by transmembrane currents (Nunez and Srinivasan, 2006). In the commonly used volume conductor theory, also used here, the extracellular medium was modeled as a smooth three-dimensional continuum with transmembrane currents representing volume current sources. The fundamental formula (Pettersen et al., 2012) relating neural activity in an infinite volume conductor to the generation of the LFP *ϕ*(*r*,*t*), at a position *r* is given by:

(1) $$\phi (r,t)=\frac{1}{4\pi \sigma }\sum_{k=1}^{n}\frac{I_{k}(t)}{\left|r-r_{k}\right|}$$

Here, *I*_k_ denotes the transmembrane current (including the capacitive current) in a neural compartment *k* positioned at *r_k_*, and the extracellular conductivity, here assumed real (ohmic), isotropic (same in all directions) and homogeneous (same at all positions), is denoted by *σ*. In the hippocampus the mean extracellular conductivity *σ* is equal to 0.3S m^– 1^ (López-Aguado et al., 2001), which is the value that we used for our simulations. A key feature of Equation 1 is that it is linear, i.e., the contributions to the LFP from the various compartments in a neuron sum up. Likewise, the contributions from all the neurons in a population would add up linearly. The transmembrane currents *I_k_* setting up the extracellular potentials according to Equation 1 were calculated by means of standard multi-compartment modeling techniques, here by use of the simulation tool NEURON Carnevale and Hines (2006). The current source densities (CSDs) in Figure 1*B* were computed using the 1D kCSD inverse method proposed in Potworowski et al. (2012). The CSDs were computed from the LFP measured by electrodes that are arranged along a straight line, in this case along the cellular axis of the PYR cell.

The same PYR cell multi-compartment model as described above was used to compute the extracellular biophysical LFP, and we used the set of 10,000 5-s raster plots (of inhibitory spikes) as described above for our presynaptic populations with the addition of basal excitation. That is, we generated extracellular potential traces (5 s each) due to the various inhibitory cell firings. We used a single multi-compartment PYR cell to compute the biophysical LFP. While an experimental LFP is generated by many cells, we still referred to our extracellular output as an “LFP” for consistency with the computational literature, where the LFP term has been used for an extracellular field from single or multiple cells.

### Simulation details

The computational simulations and analyses were performed using the LFPy python package RRID:SCR_014805 (Lindén et al., 2014), NEURON RRID:SCR_005393 (Carnevale and Hines, 2006), and MATLAB RRID:SCR_001622 (MATLAB 8.0 and Statistics Toolbox 8.1). The large scale network simulations were conducted using high-performance computing at SciNet (Loken et al., 2010). The code/software described in the paper is freely available online at https://github.com/FKSkinnerLab/LFP_microcircuit.

## Results

### Intrinsic θ rhythms in the hippocampus

It has long been known that input from the medial septum is an important contributor to *in vivo* LFP θ rhythms (Buzsáki, 2002). However, recent work by Goutagny et al. (2009) showed that θ rhythms can emerge in the CA1 region of an intact *in vitro* hippocampus preparation. These intrinsic θ rhythms appeared spontaneously without any pharmacological manipulations or artificial stimulation paradigms, and persisted even after the neighboring CA3 subfield was removed. It is thus clear that intrinsic θ frequency rhythms can be produced by local interactions between interneurons and PYR cells in the hippocampus. That is, the CA1 region of the hippocampus contains sufficient circuitry to be able to generate θ oscillations. An example of this intrinsic LFP rhythm is shown in Figure 1*B*. Considering this preparation, a one cubic millimeter estimate of the tissue size (i.e., network circuitry) needed for intrinsic θ rhythm to occur was estimated (Ferguson et al., 2013). While it is clear that these intrinsic θ rhythms do not fully encompass *in vivo* θ rhythms, they undoubtedly exist without any special manipulations, and so are arguably part of the underlying biological machinery generating θ rhythms in the hippocampus. More importantly, to have a chance to understand the many different cellular contributions to LFP recordings, this preparation can be used to decipher the many interacting components.

To examine the role of specific hippocampal interneurons in these intrinsic θ rhythms, Amilhon et al. (2015) optogenetically activated and silenced PV or SOM interneurons. PV cell types exhibiting fast firing characteristics include BCs, AACs, and BiCs (Baude et al., 2007). OLM cells are SOM-positive, but it is not the case that SOM interneurons are necessarily OLM cells. However, reconstructions of SOM cells in these studies with intrinsic θ were done, confirming that they are likely OLM cells (Huh et al., 2016). Amilhon et al. (2015) found that optogenetic manipulation of SOM cells modestly influenced the intrinsic θ rhythms. In contrast, activation or silencing of PV cells strongly affected θ. These results thus demonstrated an important role for PV cells but not SOM cells for the emergence and presence of intrinsic hippocampal θ, as given by the observed LFP recordings exhibiting θ rhythms.

LFP recordings in this preparation had a particular sink and source distribution in the different layers (Goutagny et al., 2009). It is given by a single dipole characterized by positive deflections in stratum lacunosum/moleculare (SLM) and stratum radiatum (SR) and negative deflections in SP and stratum oriens (SO). The dipole is illustrated in Figure 1*A*. This LFP laminar polarity profile was consistent across preparations. We note that since θ rhythms persisted even when the CA3 region was removed, excitatory collaterals from CA3 did not seem to be a necessity for the emergence of the rhythm and the sink/source density profile. Thus, in our LFP model in this work, we assumed that excitatory input to CA1 PYR cells was restricted to the basal dendrites due to CA1 PYR cell collaterals (Goutagny et al., 2009).

### Using a previous network model framework as a basis

To try to understand how the complex interactions between different inhibitory cell types contributed to θ LFP rhythms, a computational network framework representing CA1 microcircuitry was previously developed (Ferguson et al., 2015). Given the ambiguous role of OLM cells in θ rhythms and the newly discovered connections between OLM cells and BiCs (Leão et al., 2012), these network models were developed to explore how OLM-BiC interactions influenced the characteristics of θ rhythms. We took advantage of previously developed PV fast-firing cell models (Ferguson et al., 2013) and OLM cell models (Ferguson et al., 2015) based on recordings from the whole hippocampus preparation. Because of distal contacts of OLM cells with PYR cells, a multi-compartment PYR cell model was previously used to be able to incorporate this aspect in exploring the various interactions. The network model framework is shown in Figure 1*A*, and a summary of the network model is provided in Materials and Methods. We note that the network model was designed to explore cellular interactions and contributions to the ongoing intrinsic θ rhythms, and not to the generation of the θ rhythms explicitly. All inhibitory neurons were driven by θ frequency inputs based on experimental recordings from the whole hippocampus preparation.

As schematized in Figure 1*A*, the inhibitory cell populations encompassed BC/AACs, BiCs and OLM cells that were driven by experimentally derived EPSCs. These EPSCs were from the ongoing rhythm and were of θ frequency (Fig. 1*A*). Spiking output from the inhibitory cell populations led to IPSCs on the PYR cell. They were distributed on the PYR cell according to where the particular cell population targeted. Thus, BC/AACs to somatic regions, BiCs to middle apical and basal regions and OLM cells to distal apical regions. IPSCs generated by the different cell types are shown in Figure 1*B* (for details, see Materials and Methods). In previous work, the spatial integration of the inhibitory postsynaptic potentials at the soma of a passive PYR cell model was used as a simplistic LFP representation (Ferguson et al., 2015). This representation was in fact indicative of the intracellular somatic potential rather than the extracellular one, but it did allow the distal OLM cell inputs relative to more proximal PV cell inputs to be taken into consideration. Using this computational model framework, multiple simulations were performed and it was shown that there were parameter balances that resulted in high or low θ power, and where OLM cells did or did not affect the θ power (Ferguson et al., 2015). That is, OLM cells could play a small or large role in the resulting θ power depending on whether compensatory effects with BiCs occurred as a result of the size and amount of synaptic interactions between these cell types. Thus, interactions between OLM cells and BiCs in the CA1 microcircuitry seemed to be an important aspect for the presence of intrinsic LFP θ rhythms. However, since an *ad hoc* LFP representation was used, it was not possible to do any direct comparisons with experimentally recorded LFPs to decipher their output. That is, the possibility to parse out the contribution of the different cell types or identify particular interactions was limited. Thus, while it was possible to show that interactions between OLM cells and BiCs could play an essential role in the resulting θ power, it was not possible to predict any particular parameter balances or to extract possible explanations.

In the work here, we built on this model framework and developed biophysical LFP models. We used the inhibitory spiking output generated in Ferguson et al. (2015) as a basis for generating biophysical LFPs, and we used the same PYR cell model. However, unlike the previous work, we used the framework of volume conductor theory (see Materials and Methods) and generated actual extracellular potential output as a result of the overall activity of the inhibitory cell firings across the various layers of CA1 hippocampus. In addition, we included excitatory input onto the basal dendrites to represent recurrent CA1 inputs (see schematic in Fig. 1*A*; for details, see Materials and Methods) and directly compared with characteristics of experimental LFP recordings. It is important to note that the structure of our model here did not focus on deciphering the generation of θ rhythms directly. Rather, there was the point neuron network model with the inhibitory cells receiving θ frequency EPSC inputs and the multi-compartment PYR cell model generating biophysical LFP output based on the synaptic inputs it was receiving. In this way, we were able to do extensive parameter explorations and to focus on comparing model and experimental LFPs to gain insight.

### Overall characteristics of biophysical LFP models

From the previous modeling study of Ferguson et al. (2015), several sets of inhibitory spiking output with particular connection probabilities and particular synaptic conductances between OLM cells and BiCs were available. The connection probability from OLM cells to BiCs (*c_sb_*) varied from 0.01–0.33 with a step size of 0.02 producing 16 sets of connection probabilities; synaptic conductance values ranged from 0–6 nS for OLM cells to BiCs (*g_sb_*) and for BiCs to OLM cells (*g_bs_*) with a step size of 0.25 nS. Thus, for a given connection probability, there were 625 sets of spiking outputs from inhibitory cells, where each set represented a 850-cell inhibitory network with particular synaptic conductances. We considered a set to be a connectivity map representing the inhibitory cell populations.

For each connectivity map, we generated a biophysical, extracellular LFP. A virtual electrode probe was placed along the vertical axis of the PYR cell model to record its LFP output in a layer dependent manner. This PYR cell model was the “processor” of the LFP signal as it integrated postsynaptic inputs from different presynaptic populations. We computed LFPs at 15 equidistant sites along a linear axis (Fig. 1*A*). The PYR cell output corresponded to readouts of the postsynaptic activity elicited by the afferent inhibitory cell populations that targeted the PYR cell in appropriate regions, referred to as the LFP “generator.” We note that although there was a single connectivity map representing the randomly connected inhibitory cell population, we performed several trials when randomly targeting the PYR cell to ensure the robustness of our results (see Materials and Methods). To achieve effective electroneutrality, the extracellular sink needed to be balanced by an extracellular source, that is, an opposing ionic flux from the intracellular to the extracellular space, along the neuron; this flux was termed the “return current.”

**Figure 2.**
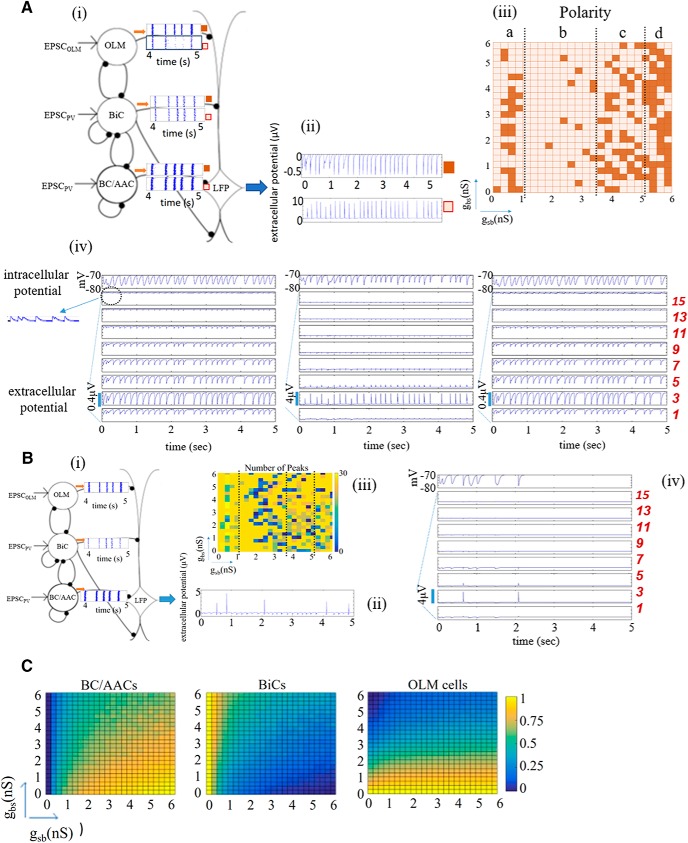
Biophysical LFP computation: features, examples, and interneuron activities. ***Ai***, Schematic shows two raster plot examples for the given inhibitory cell population rasters. ***Aii***, The resulting LFPs at the somatic layer, with positive and negative deflections is shown for the examples, labeled with dark- or light-colored squares. Parameter values are *g_sb_* = 1.5, *g_bs_* = 5.5 nS for positive and *g_sb_* = 0.5, *g_bs_* = 0.75 nS for negative deflections. ***Aiii***, The color plot on the right shows the polarity at the somatic layer, SP, electrode 4. Dotted lines delineate four regions labeled as a, b, c, and d. Negative polarity: dark-colored squares, positive polarity: light-colored squares. ***Aiv***, LFP output for all layers are shown for three examples where the polarity is negative, positive and negative at electrode 3 (left to right). Parameter values are (left to right): *g_sb_* = 0.5, *g_bs_* = 0.75; *g_sb_* = 1.5, *g_bs_* = 5.5; *g_sb_* = 5.75, *g_bs_* = 0.75 nS. Inset shows a blow up of LFP output at electrode 13 (SLM) to show positive deflections. Also shown is the intracellular somatic potential of the PYR cell. No basal excitation is present, *c_sb_* = 0.21. ***Bi***, Schematic includes one raster plot example. ***Bii***, The resulting LFP output at SP has five peaks. A maximum of 29 peaks is possible (see text). Parameter values are *g_sb_* = 2, *g_bs_* = 0.75 nS. ***Biii***, The color plot shows the number of peaks that appear in the 5-s LFP computation at SP, electrode 4. Dotted lines delineate the same regions as in ***A***. ***Biv***, An example of LFP output for all layers as well as the intracellular somatic output which also shows a loss of peaks. Parameter values are *g_sb_* = 2.25, *g_bs_* = 5.0 nS. No basal excitation is present, *c_sb_* = 0.21. ***C***, Interneuron activity for each interneuron population, normalized such that the number of spikes for a given pair of synaptic conductances is divided by the maximal number considering all pairs of synaptic conductances. Maximal number (5-s trace): 16 327 (BC/AACs), 6 808 (OLM cells), 4 589 (BiCs).

We developed some initial intuition regarding the generation of our biophysical LFPs by computing them without including basal excitation. That way, all of the inputs received by the PYR cell model were inhibitory. Figure 2*A*,*B* illustrates the process and shows some examples. Let us first focus on Figure 2*Ai*. Next to each cell population in the network schematic are two examples of 1-s raster plots of spiking outputs (from the previously computed 5-s inhibitory network simulations in Ferguson et al., 2015) produced for particular parameter sets. These spikes gave rise to IPSCs on the PYR cell model and the computed extracellular LFP at the somatic layer is shown in Figure 2*Aii*. As shown, these particular parameter sets produced LFPs with positive or negative deflections. Let us next focus on Figure 2*Bi*. One example of a 1-s raster plot is shown, and for this parameter set, the LFP had only a few positive deflections, as shown in Figure 2*Bii*. Assuming that one population burst in the raster plot leads to a single peak in the LFP, there would be ∼29 peaks in the LFP for a 5-s simulation (i.e., ∼5.8-Hz frequency), since our inhibitory cell raster plots have 28–29 population bursts. Note that the raster plots in Figure 2*Bi* were not very different from the examples shown in Figure 2*Ai*. We computed LFPs at all layers as represented by the 15 virtual electrodes shown in Figure 1*A* for the 625 sets of inhibitory spiking outputs across *g_sb_* and *g_bs_* values at a particular connection probability *c_sb_*. The colored plot in Figure 2*Aiii* shows the polarity of the LFPs at the somatic layer, and the color plot in Figure 2*Biii* shows the number of LFP peaks in the somatic layer. In Figure 2*C*, normalized spike numbers for all interneuron populations are shown.

As a first approximation, given the network model framework and previous work, we can say the following about the LFPs: those governed mainly by synaptic inputs and not return currents were characterized by narrow wave form shapes as the synaptic inputs from any particular interneuron population enters the PYR cell in a synchronized fashion. This was due to the inhibitory cells in a given population being driven by rhythmic EPSCs that gave rise to coherently firing inhibitory cells in a given population (see example raster plots). We note that the EPSCs that were used in the simulations were not perfectly synchronized since the measured experimental variability was included in designing the EPSC inputs to use in the inhibitory network simulations (see Materials and Methods). On the other hand, return currents constituted a summation of less synchronized exiting currents that originally entered the cell at different locations. Therefore, LFP deflections governed by return currents were generally wider. Further, we would expect that the LFP recorded from different layers would first and foremost be influenced by the interneurons that project to that region. We also note that the width of the LFP deflection would not only be influenced by the nature of the current (synaptic inputs or return currents) but also by the synaptic time constants defining the shape of the IPSCs. IPSCs for the different cell populations are shown in Figure 1*B*, where it can be seen that the IPSCs produced by OLM cells and BiCs were wider relative to the IPSCs from BC/AACs. Thus, we expected that positive LFP deflections would be recorded in locations where OLM cells, BiCs, and BCs project, with wider LFPs for OLM cell projection locations, and that LFPs dominated by return currents would be recorded in locations where there were no direct inputs from interneurons. However, due to interactions between BiCs and OLM cells, this was not necessarily the case as return currents from distant interneuronal inputs could prevail in regions where other interneurons directly projected. In fact, interactions between OLM cells and BiCs can strongly modulate the relative balance between synaptic inputs and return currents, which in turn can strongly modulate the distribution of sinks and sources in the resulting LFP.

The two examples of LFP output at the somatic layer in Figure 2*Aii* show one with narrow positive deflections and the other with wider negative deflections. This thus indicated that the BC/AAC inputs that synapse at the somatic layer dominated for the positive deflection LFP example whereas BiC and OLM cell inputs that synapse more distally dominated for the negative wider deflection LFP example. The example in Figure 2*Bii* of LFP output at the somatic layer indicated that a loss of peaks can occur due to the superposition of synaptic inputs and return currents. Another “loss of peaks” example is shown in Figure 2*Biv*, and LFP output from multiple layers is shown in addition to the intracellular somatic output. For this example, the peak loss was also partially reflected in the intracellular somatic output. However, loss of peaks in the LFP output was not necessarily reflected in the somatic intracellular recording. Note that since the PYR cell was only receiving inhibitory input in these set of simulations, somatic intracellular potentials always had negative deflections. How the extracellular potential features changed as a function of the synaptic conductances between BiCs and OLM cells is summarized in the color plots of Figure 2*Aiii* for the polarity and Figure 2*Biii* for the number of peaks (somatic layer).

Let us consider Figure 2*Aiii*. We found that we can approximately distinguish four regions as *g_sb_* was increased. These regions are separated by dotted lines in Figure 2*Aiii* and labeled *a* to *d*. For small *g_sb_* values (0–1 nS, region *a*) the amount of inhibition that the BiCs received from the OLM cells was minimized allowing the BiCs to be at the peak of their activity (Fig. 2*C*). Consequently, the inhibition that the OLM cells and BC/AACs received from the BiCs was maximized causing their activities to be minimized (Fig. 2*C*). As a result, the extracellular potential in the somatic region was governed by return currents leading to negative polarity LFPs in the somatic layer (i.e., mainly dark-colored in region *a* of Fig. 2*Aiii*), primarily due to the BiC synaptic inputs on the “middle” region (SR layer) and “basal” region (SO layer) of the PYR cell. As we increased *g_sb_* (1–3.5 nS, region *b*), we encountered mainly positive polarity LFPs (i.e., light-colored in region *b* of Fig. 2*Aiii*). In region *b*, the inhibition onto the BiCs was increased and thus their activity was decreased, as can be seen in Figure 2*C*, causing a decrease in the amount of the inhibitory current onto the PYR cell from BiCs. As a result, the magnitude of the return currents caused by the BiC synaptic inputs was decreased at the somatic layer. Simultaneously their ability to inhibit the BC/AACs was also decreased so that the BC/AACs became more active and their direct inhibition onto the PYR cell also increased. Since both BiCs and OLM cells activity was low in region *b*, while BC/AAC activity was increased, the somatic LFP was governed by BC/AAC inputs rendering the extracellular LFP positive. As we further increased *g_sb_* (3.5–5 nS, region *c*) the silencing of the BiCs increased even further and their ability to silence the BC/AACs was further reduced. Simultaneously OLM cell activity increased. Thus, the somatic LFP was influenced by direct synaptic inputs from BC/AACs and also return currents from OLM cells (sparse dark-coloring region *c*). Interestingly, the majority of the loss of peaks in somatic LFP output occurred in regions *b* and *c* (see blue-green pixels in the Fig. 2*Biii*), where superposition of synaptic inputs and return currents was mostly occurring. That is, cancellations occurred even leading to abolishment of the entire rhythm sometimes. Finally, for *g_sb_* from 5.0–6 nS (region *d*), the BiCs were maximally inhibited and BC/AACs were at the peak of their activity. While we might have expected domination from the BC/AAC synaptic inputs for these values, it turns out that return currents (negative polarity) dominated. This can be explained by the increased activity of OLM cells which were also at the peak of their activity producing strong return currents in the somatic region. In summary, light-colored regions in Figure 2*Aiii* signify that BC/AACs dominated the extracellular somatic potential and dark-colored regions signify that other inhibitory cell types (BiCs or OLM cells, or both) contributed more strongly.

In Figure 2*Aiv*, we show three examples of LFP recordings at multiple layers as well as the somatic intracellular potential, for increasing values of *g_sb_* from left to right. To allow an appreciation of the changing magnitude of the signal, we used the same resolution on the ordinate axis for all LFP plots shown. On the left (*g_sb_* = 0.5 nS) we see that the signal was governed by return currents (negative polarity) in the entire SP (electrodes 3 and 5), in SO (electrode 1), and in SR (electrodes 7, 9, and 11). Synaptic events governed SLM (electrodes 13 and 15) where OLM cells directly project leading to positive polarity. In the middle (*g_sb_* = 1.5 nS), the LFP in SP and SO was governed by synaptic inputs (positive polarity), and in SR and SLM by return currents (negative polarity). As expected, we found that the positive polarity LFP in SP here was narrower relative to the positive polarity LFP in SLM on the left, because the IPSCs produced by OLM cells were wider relative to those of BC/AACs, as shown in Figure 1*B*. On the right where *g_sb_* = 5.75 nS, we observed a similar trend as for the example on the left where *g_sb_* = 0.5 nS with return currents dominating.

We would like to use our computational LFPs to determine how the different inhibitory cell types contributed to θ LFPs as recorded experimentally in the *in vitro* whole hippocampus preparation. As described above, our overall network model (Fig. 1*A*) was intended to capture an intrinsic θ rhythm in the CA1 region of the *in vitro* preparation. CA3 input was not required but local excitatory input which occurs on basal dendrites (Takács et al., 2012) did need to be included. To do this, we took advantage of previous modeling studies (Bezaire and Soltesz, 2013; Ferguson et al., 2015) as detailed in Materials and Methods. Including excitatory input would clearly affect resulting biophysical LFP outputs. Specifically, the LFP amplitude in SO might decrease even further in the presence of basal excitation as excitatory and inhibitory BiC inputs could cause mutual cancellations in this region. As return currents mostly exit close to the somatic region where the surface area is larger, the effect of basal excitation might be stronger in SO and SP since most of the current might have exited before reaching SR and SLM. In general, we expect there to be a range of possible LFP characteristics based on the above LFP computations done in the absence of basal excitation. We expect that the addition of excitatory input will influence the LFP in non-intuitive and nonlinear ways and the intuition developed above will be helpful in deciphering and explaining the contribution of the different cell populations to the LFP.

### Constraining synaptic conductances and connection probabilities between BiCs and OLM cells

In this work, we focused mainly on OLM cells. The previous model network framework (Ferguson et al., 2015) was developed based on knowing that connections exist between BiCs and OLM cells (Leão et al., 2012). Given this, there were two pathways to consider for how OLM cells could influence ongoing intrinsic θ LFP rhythms. They can influence LFP output indirectly through disinhibition of proximal/middle dendrites of the PYR cell (OLM-BiC-PYR, indirect pathway), or directly through inhibition of distal, apical dendrites of the PYR cell (OLM-PYR, direct pathway). As shown above, many different LFP features can be exhibited in the absence of basal excitation (Fig. 2*A*,*B*). It is interesting to note that our biophysical LFP output did not necessarily exhibit θ frequencies, despite being driven by θ frequency EPSC inputs (Fig. 2*Bii*). This is because cancellations in the extracellular space between synaptic inputs and return currents can result in loss or even abolishment of the rhythm. This underscores the importance of modeling biophysical LFPs as the interaction of synaptic and return currents on the extracellular signal can strongly affect the resulting LFP frequency.

**Figure 3.**
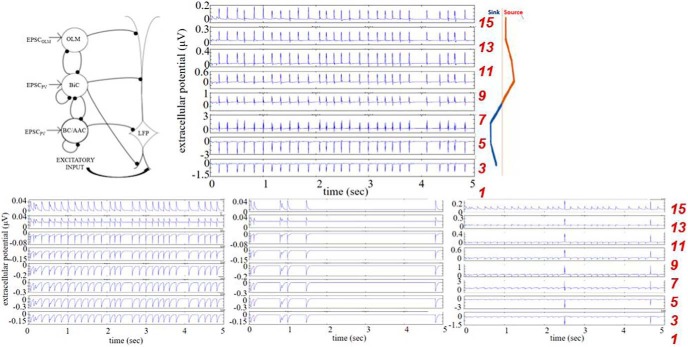
Example LFPs from selected and rejected parameter sets. Computed LFPs are shown across multiple layers. Top, Selected parameter set: *g_sb_* = 6, *g_bs_* = 1.25 nS. Bottom, Rejected parameter sets (left to right): *g_sb_* = 0.5, *g_bs_* = 0.75 nS; *g_sb_* = 0.5, *g_bs_* = 3.5 nS; *g_sb_* = 2.5, *g_bs_* = 1 nS; *c_sb_* = 0.21 for all.

We proceeded to include basal excitation and performed a full set of computations for all connection probabilities (*c_sb_*) and synaptic conductances (*g_sb_*, *g_bs_*). With these computed biophysical LFPs in hand, we did direct comparisons with experimental LFPs from the whole hippocampus preparation *in vitro.* Specifically, we classified each set of network parameters as selected or rejected based on whether our computed LFPs were able to reproduce two robust characteristics exhibited experimentally. These were: (1) the laminar polarity profile exhibited a single dipole with sinks in the basal dendrites and sources in the apical dendrites, and (2) the frequency of the LFP traces across all layers was in the θ frequency range. These characteristics are shown in Figure 1*A*. We note that our model setup in which experimentally derived θ frequency EPSCs were input to the inhibitory cells means that the LFP rhythm should have a θ frequency. However, as we have shown above, the resulting biophysical LFP frequency can be much less than θ due to synaptic and return current interactions and cancellations (Fig. 2*Bii*). Specifically, the frequency of the EPSCs used from experiment is ∼5.8 Hz. Thus, in enforcing the θ frequency on our LFP computations, it was only necessary to impose a lower bound. We used 3 Hz as the lower bound for θ range to be similar to experiment (Goutagny et al., 2009). We applied a peak detection on the LFP trace and used a threshold to avoid detecting baseline peaks. We required that the number of peaks be larger than 15 which given the 5-s LFP trace corresponds to 3 Hz. In Figure 3, top, we show an example of computed LFPs across the different layers for a parameter set that was selected. The bottom of Figure 3 shows LFP outputs for three different parameter sets that were rejected - incorrect polarities and frequencies are apparent. Note that ordinate resolutions were adjusted across the layers so that the frequency and polarity of computed LFPs can be readily seen in each layer in viewing.

**Figure 4.**
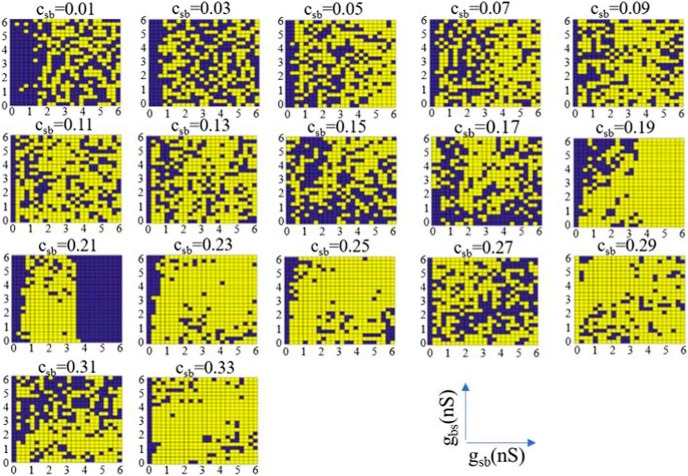
All selected and rejected parameter sets. Parameter sets are considered as selected (purple) if computed LFPs match LFPs from experiment in polarity and frequency (3-Hz lower bound). Otherwise, as rejected (yellow). A clear separation in parameter space occurs for *c_sb_* = 0.21.

**Figure 5.**
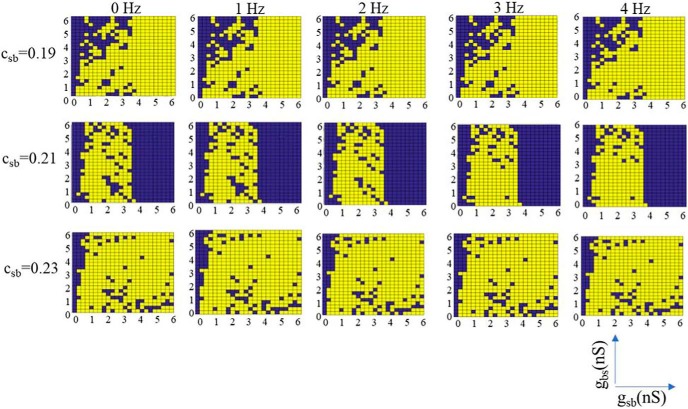
Selected and rejected parameter sets using different lower frequency bounds. The different frequency bounds used are shown at the top of each column and only three different *c_sb_* values are shown. Note that we use 3 Hz as the frequency bound in Figure 4.

Classifying each parameter set, we summarize our results in Figure 4, where selected parameter sets are shown in purple and rejected ones in yellow. We observed the following: for low *c_sb_*, the plots have a checkered appearance since small changes in *g_sb_* and *g_bs_* caused the system to alternate between being selected or rejected. As *c_sb_* increased, there was a clearer separation in (*g_sb_*, *g_bs_*) parameter space of selection or rejection. This was observed from *c_sb_* = 0.19 to *c_sb_* = 0.25. In this range, we considered the system to be robust as it was not very sensitive to synaptic conductance perturbations. However, for *c_sb_* = 0.19, 0.23, and 0.25, the selected parameter sets were quite narrow. As *c_sb_* was further increased, the checkered patterning returned. Note that the selected sets were mainly affected in one direction as *c_sb_* changed. That is, across *g_sb_* rather than *g_bs_* values. Further, we note that in doing this classification, it was more the polarity criteria rather than the frequency criteria of the LFP signal that delineated selected and rejected parameter sets. This is shown in Figure 5, where we did not apply any frequency bound or used different lower frequency bounds. While there was some change in selected and rejected parameter sets, they were minimal.

**Figure 6.**
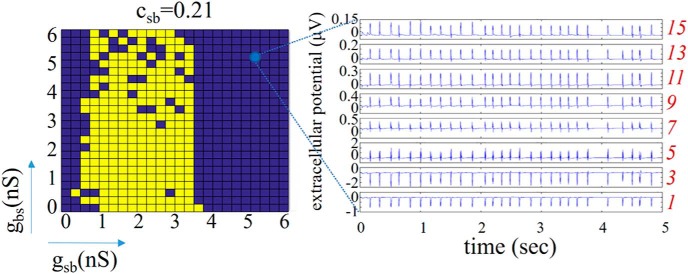
Predicted regime. For *c_sb_* = 0.21, selected parameter sets (purple) include g_sb_ values of 3.5–6 nS, and all *g_bs_* values. Rejected sets are in purple. On the right are LFP traces from 8 electrodes for a parameter set of *g_sb_* = 4.75, *g_bs_* = 4.50 nS.

Since there is natural variability in biological systems, we assumed that sensitivity to small perturbations in parameter values is anathema to having robust LFP θ rhythms. Noting that the synaptic conductance resolution in our simulations was 0.25 nS, and that a minimal synaptic weight can be estimated as larger than this (see Materials and Methods), we considered that (*g_sb_*, *g_bs_*) parameter sets that did not yield at least two complete, consecutive rows or columns of purple (selected) were inappropriate for the biological system. That is, variability that was less than a minimal synaptic weight would not make sense. Looking at this in Figure 4, we first note that there were never at least two complete purple rows for any *c_sb_*, but there were cases of two or more complete purple columns, namely, *c_sb_* = 0.03 and 0.21. However, a complete purple column for *g_sb_* = 0 was invalid since it is known that OLM to BiC connections exist Leão et al. (2012). Thus, *c_sb_* = 0.03 can be eliminated leaving *c_sb_* = 0.21 as appropriate. For this connection probability, the transition from selected to rejected networks and vice versa strongly depended on *g_sb_* rather than on *g_bs_* values, revealing a more important role for the former. In summary, by directly comparing characteristics of our computed biophysical LFPs with those from experiment, we were able to constrain an appropriate connectivity as *c_sb_* = 0.21, with *g_sb_* values of 3.5–6 nS, and the full set of *g_bs_* values (*g_sb_* ≠ 0, *g_bs_* ≠ 0). We will refer to this set of parameter values as the predicted regime. In Figure 6, we show example LFP responses across several layers for a set of parameter values from this predicted regime.

### OLM cells ensure a robust θ LFP signal, but minimally affect LFP power, and only through disinhibition

**Figure 7.**
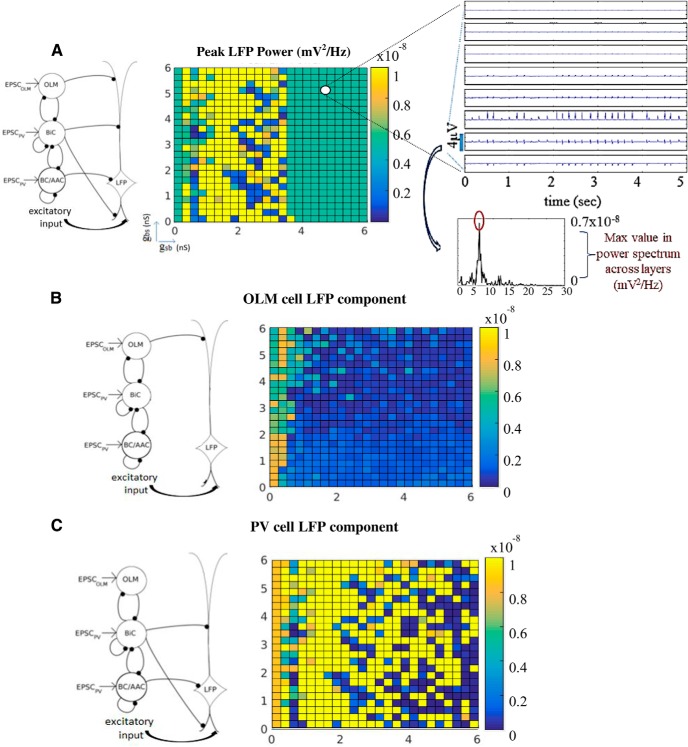
Decomposition of the LFP signal. ***A***, All presynaptic cell populations are present. ***B***, Only OLM cells are present. ***C***, Only BiCs and BC/AACs are present. Schematics on the left show the cell populations projecting to the PYR cell. Computations are done across *g_sb_* and *g_bs_* parameter values where *c_sb_* =0.21. For each parameter set, LFPs are computed across all layers and the power spectrum is computed for each layer. The maximum power across all layers is taken as the peak power and given in the color plot. Computation is illustrated to the right of ***A*** (see text for details).

**Figure 8.**
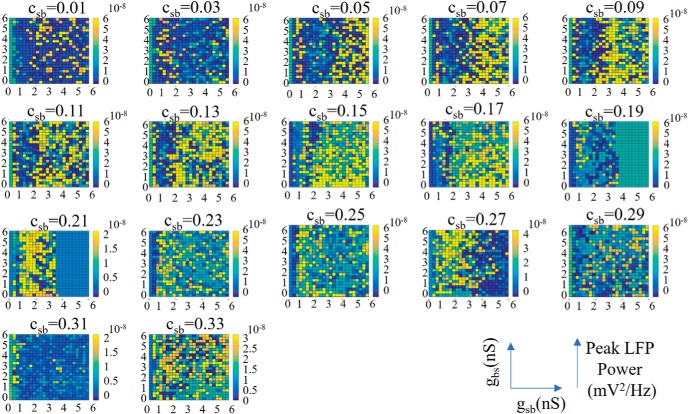
Peak power for all conductances and connectivities. Note that the color scale bars are not the same for all the plots. The plot for *c_sb_* = 0.21 corresponds to Figure 7*A*.

In continuing our analysis, we now focused on constrained parameter sets as determined above which we termed the predicted regime (*c_sb_* = 0.21). We decomposed the signal to be able to examine the contribution of the interneuron subtypes to the power of the LFP. We separated our interneuron subtypes into two groups: PV subtypes which are BC/AACs and BiCs, and SOM subtypes which consist of the OLM cells here. These two groups were represented by distinct mathematical models of fast-firing PV and SOM inhibitory cells based on whole-cell recordings from the whole hippocampus preparation (Ferguson et al., 2015). We performed spectral analyses of our computed LFPs and used the peak amplitude as a measure of the power of the θ network activity. The peak power was computed for each of the 15 electrodes (i.e., all layers), and we plotted the maximum value from all of the layers in the color plots of Figure 7. This is illustrated on the right of Figure 7*A*. We first simulated the spectral LFP power when all presynaptic inhibitory cell populations were present. As shown in Figure 7*A*, a robust power feature emerged. When all presynaptic origin populations were present, the predicted regime shown in purple in Figure 6, produced LFP responses whose power showed minimal variability. This is an interesting observation on its own, as the power of the LFP varied little across hippocampus preparations (Goutagny et al., 2009). Thus, our predicted regime satisfied another characteristic of experimental LFPs. We note that outside of the predicted regime, the LFP output showed much more variability, and the LFP frequency across layers was not necessarily θ, as it was not part of the selected parameter sets. For completeness, we show peak power computations that were done for all connectivities in Figure 8.

To examine the role of presynaptic origin populations on the LFP we decomposed the signal by selectively removing OLM to PYR cell connections or PV to PYR cell connections and then computing and plotting the peak power as described above. Selective removal of synapses from PV cells to the PYR cell yielded an LFP response whose presynaptic origin population was due to the OLM cell population. The resulting LFP power was low and depended weakly on *g_bs_* (Fig. 7*B*). This showed that OLM cells minimally contributed to the signal power as a presynaptic origin population. Viewing this from a broader perspective, these results indicated that disinhibition of non-distal apical dendrites via an indirect (OLM-BiC-PYR) pathway played a much larger role relative to a direct (OLM-PYR) pathway in producing the LFP power. Along the same lines, disinhibition of distal dendrites through a BiC-OLM-PYR pathway thus did not have much of an effect on LFP power. Figure 7*C* shows the result when we selectively removed the synapses from OLM cells to the PYR cell to yield an LFP response whose presynaptic origin was the PV cell population. It is clear from the magnitude of the signal powers in Figure 7*C* relative to Figure 7*B* that the θ power was indeed mainly due to the component from PV cells rather than from OLM cells. Interestingly, the previously seen robustness when all presynaptic cell populations were present (Fig. 7*A*) was now lost. To quantify all of this, we computed the mean and SD of the peak powers in the predicted regime for Figure 7*A–C*. Respectively, they were (mean, SD) in units of *mV* ^2^∕*Hz*: (5.1 × 10^– 9^, 1.7 × 10^– 23^), (9.7 × 10^– 10^, 5.6 × 10^– 10^), (2.6 × 10^– 8^, 3.8 × 10^– 8^). When all of the cell populations were present, there was minimal variability, and when the PV cell populations were removed, the average power decreased five-fold and there was some variability. However, when only PV cell populations were present, there was an increase in the average power and the variability was large. It seems clear that OLM cells did not contribute much to the average LFP power but removing their inputs prominently affected the robustness of the LFP signal. Therefore, we propose that OLM cells have the capacity to regulate robustness of LFP responses without affecting the average power.

In a recent study, Amilhon et al. (2015) showed that SOM cells (putative OLM cells) did not appear to play a prominent role in the generation of intrinsic LFP θ rhythms since there was only a weak effect on LFP θ power when they optogenetically silenced SOM cells. Our results are in agreement with this observation. As shown in Figure 7*B*, the contribution of OLM cell inputs to the LFP power was small. To make a more accurate comparison with Amilhon et al. (2015)’s OLM cell optogenetic silencing experiments, we compared the power of the LFP in the predicted regime in Figure 7*A* (mean value of 5.1 × 10^– 9^ *mV* ^2^∕*Hz*) with the power of the LFP in Figure 7*C* for *g_sb_* = 0 and *g_bs_* = 0 when OLM cell to PYR cell connections were also removed (8.5 × 10^– 9^ *mV* ^2^∕*Hz*). They were clearly comparable. It is interesting to note that it was already apparent from Figure 7*A* that OLM cells minimally affected LFP power. Consider that for the parameter regime of *g_sb_* = 0 and across all *g_bs_*s, the LFP power magnitude was the same (5.1 × 10^– 9^ *mV* ^2^∕*Hz*) as the average power of the predicted regime in Figure 7*A*. In this *g_sb_* = 0 parameter regime, OLM cell to BiC connections were not present but the OLM cell to PYR cell connections were still present so that OLM cells could still contribute to the LFP response via a direct OLM-PYR pathway. Given that the power did not change indicates that any LFP power contribution due to OLM cells occurred mainly via the indirect OLM-BiC-PYR pathway. Overall, our results show that OLM cells did participate but in such a way that their presence would be unnoticed if one were only measuring LFP power.

**Figure 9.**
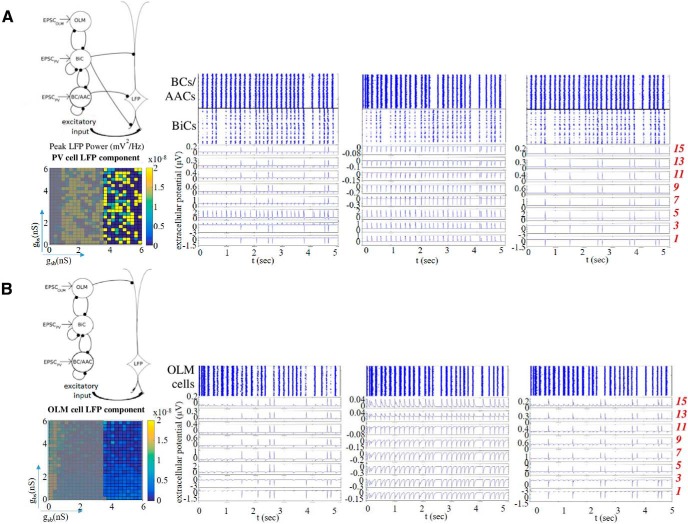
LFP pattern examples in predicted regime when only either PV or OLM cell populations are present. Peak power color plots as in Figure 7 are shown but with a different color resolution. A gray overlay is added to the plots to emphasize the predicted regime. Three examples of LFP responses (5 s) across the different layers are shown to illustrate the different patterns observed. For each example, spike rasters for the particular example are shown for PV cells (BiCs and BC/AACs) or OLM cells. ***A***, PV cell LFP component. ***B***, OLM cell LFP component. Parameter values for left, middle and right columns are, respectively, (*g_sb_*, *g_bs_*) = (5, 2.75), (5.5, 0.5), (5.75, 1) nS.

To gain insight into how OLM cells affected the robustness of the LFP signal, we further examined what was revealed with our LFP decompositions. We observed that with PV or OLM cells removed, the impaired LFP output could be grouped into certain categories based on their laminar LFP profiles. In Figure 9, we show the peak power plots for the PV cell (Fig. 9*A*) and OLM cell (Fig. 9*B*) decomposition components in which the non-predicted regime was overlaid with gray. For each component, we show three examples of the characterized LFP profiles identified in the groupings. Raster plots that corresponded to each cell population are shown above the examples in the figure. It is evident that the different LFP patterns cannot be intuited from the raster plots alone. These examples illustrate the various cases of impaired LFP responses that occurred when OLM or PV cell connections to the PYR cell were removed.

For the middle LFP response examples (low *g_bs_* and high *g_sb_*) of Figure 9, we note that OLM cells and BC/AACs had maximal activities and BiCs had minimal activities (Fig. 2*C*). Thus, synaptic current influences were obvious at the layers where OLM or BC/AACs contact, and return currents at other layers. Inappropriate polarity across the layers was manifest. This pattern of impaired LFP response occurred in about a quarter of the PV cell LFP component parameter sets, and in less than half of the OLM cell LFP component parameter sets. For the PV cell LFP component, most of the other parameter sets yielded LFP responses in which there was no rhythm, as shown in the right example of Figure 9*A*. Interestingly, in the rest of the cases (less than a third), there was a loss of rhythmicity in all layers except for the somatic layer as illustrated in the left example. These patterns show that there was an ongoing “battle” between basal excitation and PV cell inputs that can yield a wide range of LFP powers from low (no rhythm, right example) to high (left and middle examples). For the majority of the OLM cell LFP component parameter sets, there was a loss of rhythmicity as shown in the left and right examples of Figure 9*B*. From the temporal profile and polarity, it was clear that the high amplitude LFP peaks were due to basal excitatory inputs. For larger *g_bs_* values, OLM cells were less active (Fig. 2*C*) and LFP responses across the layers became dominated by peaks due to basal excitation rather than synaptic and return currents due to OLM cells. Overall, cancellations and rhythm loss occurred due to interactions between OLM cells’ synaptic and return currents and excitatory inputs. As summarized in the peak power plots of Figure 7*C* or Figure 9*A*, PV cell inputs alone were not capable of sustaining the robustness throughout the predicted regime and the impaired LFP signals showed a large variability. With OLM cell inputs alone, there was low LFP power either because of loss of rhythmicity or because of low amplitude rhythms (Fig. 7*B* or Fig. 9*B* peak power plots).

### With and without basal excitation

**Figure 10.**
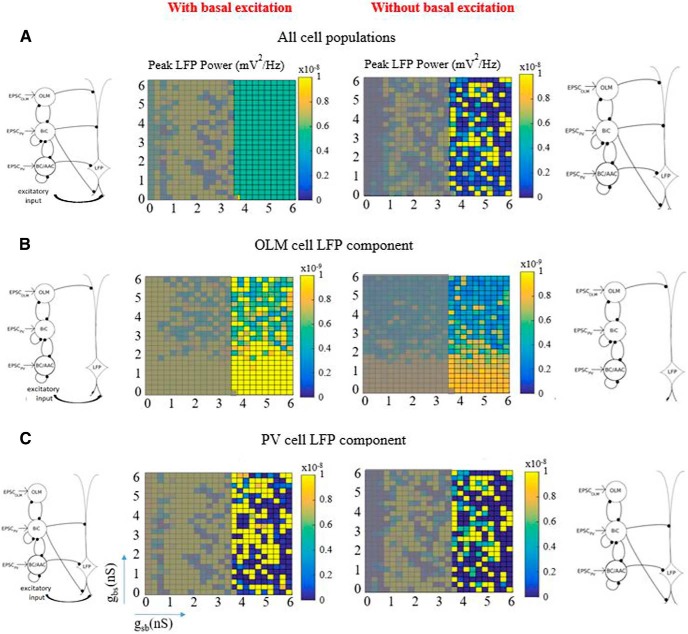
Peak power plots with and without basal excitation. The color plots represent peak power as described in Figure 7 and with a gray overlay as in Figure 9. Note that different color resolutions are used here to facilitate comparison for particular cell populations (i.e., any row). With and without basal excitation is shown on the left and right columns, respectively. Top, All cell populations. Middle, OLM cell LFP component. Bottom, PV cell LFP component.

As one might expect, including basal excitation to incoming inhibitory inputs from different cell populations added to the complexity of untangling nonlinear, interacting components producing the LFP. We relied on our developed intuition when basal excitation was not included (Fig. 2*A*,*B*) and our LFP decompositions to help reveal the different roles that OLM cells and PV cells might play in LFP θ rhythms. Specifically, we can understand that the loss of LFP rhythm at some layers likely occurred because of having a “balance” of synaptic and return currents for various conductance values leading to LFP rhythm cancellation or an inappropriate negative polarity domination (Fig. 2*Aiii*,*Biii*). Thus, in finding that the LFP power was a robust feature in the predicted regime of synaptic conductance and connection probabilities, we were able to understand that it was critically the OLM cell population that brought about this robust feature. However, this robust feature was apparent only when basal excitation was included. This is clearly visualized in Figure 10, where we plot the peak power color plots with and without basal excitation when all cells were present or with only OLM cell or PV cell LFP components. Removal of basal excitatory inputs in the case when all cells were present (Fig. 10, top) led to a loss of robustness. The mean and SD in the predicted regime without basal excitation was 6.2 × 10^– 9^ and 8.0 × 10^– 9^ *mV* ^2^∕*Hz*, respectively. While the mean was comparable to when basal excitation was present, the SD was much larger (see values with basal excitation above). Coactivation of inhibition and excitation was clearly important for this robust feature to emerge.

From the LFP decompositions and different LFP patterns expressed (Fig. 7*B*), and OLM cell activities (Fig. 2*C*), we can understand that the contribution of OLM cells was more dependent on *g_bs_* than *g_sb_* with the basal excitation affecting the peak power robustness more for larger *g_bs_* values. This was apparent in the color variation of the plots of the OLM cell LFP component in Figure 10, middle. It was larger with basal excitation (left) than without basal excitation (right) for larger *g_bs_* values. This was reflected in the mean and SD without basal excitation (5.2 × 10^– 10^, 2.2 × 10^– 10^ *mV* ^2^∕*Hz*) that was smaller than with basal excitation (see values with basal excitation above). With only the PV cell LFP component, the LFP θ rhythm was disrupted as the interactions between basal excitation and PV inhibitory inputs were missing the OLM cell inputs. Specifically, the mean and SD without basal excitation was (8.0 × 10^– 9^, 1.1 × 10^– 8^ *mV* ^2^∕*Hz*) which was smaller than with basal excitation (see values with basal excitation above). In essence, the inclusion of basal excitation can be considered as “adding” to the magnitude and variance of the LFP power when OLM cells or PV cells were examined separately. In combination, a synergistic effect between inhibition and excitation occurred to generate a robust regime, a mean power with minimal variance. From Figure 2*C*, it can be seen that the PV cells (BC/AACs and BiCs) had activities that were more dependent on *g_sb_* than on *g_bs_*, and that BC/AACs were relatively more active than BiCs in the predicted regime. Thus, at larger *g_bs_* values when OLM cells were less active, BC/AACs would contribute more to keeping a synergistic balance with the basal excitation.

### LFP power across layers

**Figure 11.**
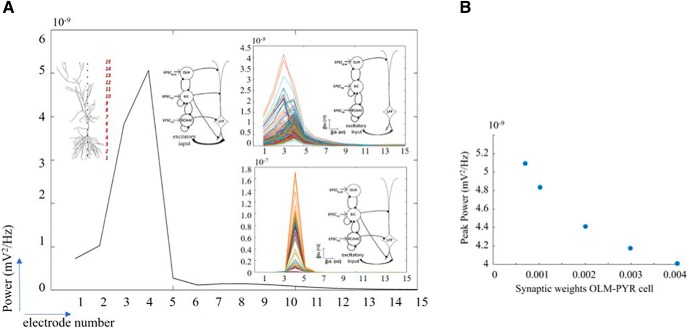
Laminar power and peak power changes with changing synaptic weights. ***A***, Computed power at the different electrode locations to show laminar power distribution, for all sets of parameter values in the predicted regime. Top inset, Laminar power for OLM cell LFP component. Bottom inset, Laminar power for PV cell LFP component. Schematics shows the PYR cell model with the 15 extracellular electrodes and the different network configurations. ***B***, Changing the synaptic weight from the OLM cells to the PYR cell does not lead to much change in the peak power, as illustrated by the peak power at electrode 4. Parameter values: *g_sb_* = 5.25, *g_bs_* = 5.00 nS. Synaptic weights of 0.00067, 0.001, 0.002, 0.003, and 0.004 μS are shown.

As illustrated in Figure 7*A*, the color peak power plots are the power in the layer (particular electrode) where the power was maximal. To fully express this, we plotted the maximum LFP power across the dendritic tree for all parameter sets in the predicted regime. This is shown in Figure 11*A* with insets showing the same for the OLM cell (top) and PV cell (bottom) LFP components. From this, we see that the maximum LFP power was recorded at electrode 4, and that with only the OLM cell component, the power was distributed more widely and with only the PV cell component, more narrowly focused around the soma. This thus shows that the two populations differentially influenced the location of LFP maxima. That the LFP power showed no discernible variability when all the cell populations were present, and that there was clear variability when not all of the cell populations were present is obvious in this Figure 11*A*. We did several additional sets of simulations to explore whether changes in the synaptic weights on the PYR cell would affect whether the robust power feature in the predicted regime would still be present. In all the simulations presented so far, we used synaptic weights that did not bias the effect of one cell population type over the other based on their synaptic input location. So, for example, OLM cell inputs that were the furthest away from the soma had the largest synaptic weight. In doing this, we were following what was done previously in Ferguson et al. (2015) who used “unbiased” synaptic weights as well as using the same synaptic weight for all of the cell types. In using the same synaptic weight for all the cell types, we found that the robust power feature in the predicted regime remained (data not shown).

As described and shown above, it was already clear that OLM cells via a direct OLM-PYR pathway minimally contributed to the LFP θ power. To show this directly, we did several, additional simulations where we changed the synaptic weight from OLM cells to the PYR cell. As an example, in Figure 11*B*, we show that increasing the synaptic weight by almost an order of magnitude decreased the peak power by only ∼20%.

### Estimating the number of PYR cells that contribute to the LFP signal

It is challenging to know how many cells contribute to an extracellular recording. The hippocampus has a regular cytoarchitecture with a nearly laminar, stratified structure of PYR cells (Andersen et al., 2006). This arrangement together with PYR cells being of similar morphologies and synaptic input profiles means that we can assume that any given PYR cell will generate a similar electric field leading to an additive effect in the extracellular space with multiple cells in resulting LFP dipole recordings. Further, for the *in vitro* intrinsic θ LFP generation being considered in this work, the focus can be justified to the couple of synaptic pathways that we explored, and incoming inputs were synchronized amplifying the additive effect.

**Figure 12.**
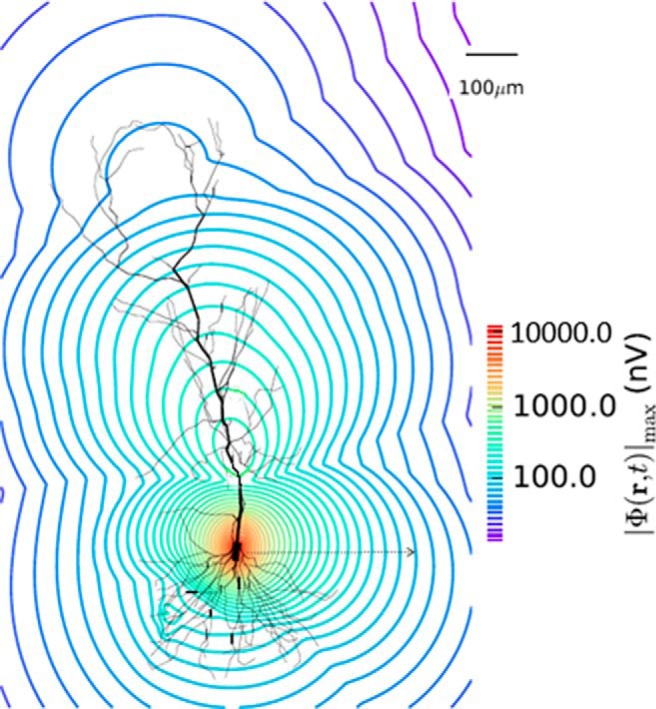
Spatial attenuation. We estimated the spatial extent of the generated LFP using our models. PYR cell model morphology is shown with calculated signal decrease from an electrode positioned near the cell soma. The dotted arrow shows the extent of the spatial reach of the signal that is taken as a 99% decrease in the signal, and is ∼300 μm. Parameter values used are from the predicted regime; *g_sb_* = 5, *g_bs_* = 5.75 nS, *c_sb_* = 0.21.

To estimate how many PYR cells contributed to an extracellular LFP recording in the *in vitro* whole hippocampus preparation, we defined the “spatial reach” of the LFP as the radius around the electrode where the LFP amplitude was decreased by 99%. Using our biophysical computational LFP models with parameter values taken from the predicted regime, we found that the spatial reach is 300 μm as measured extracellularly close to the soma since the LFP decreased from 10,000 to 100 nV within this radius. This is shown in Figure 12, where the dotted arrow represents this radius. Therefore, from a “neuron-centric” approach the LFP declined to 1% of its original power within 300 μm. From an “electrode-centric” point of view this means that if we were to place an electrode extracellularly to the soma of a given neuron then that electrode would pick up signal from neurons within 300 μm as any neuron 300 μm further away would contribute to the recorded signal by <1% of its maximum power. To estimate the number of cells present within this spatial extent we turned to literature. Taking advantage of detailed quantitative assessment and modeling done by Bezaire and colleagues (Bezaire and Soltesz, 2013; Bezaire et al., 2016), there are ∼311,500 PYR cells in a volume of 0.2 mm^3^ of “SP” tissue (see model specifics in Bezaire et al., 2016, their Fig. 1). Given our spatial reach radius estimate, a cylindrical volume of SP would be 0.014 mm^3^ or ∼7% of the total number of PYR cells which is ∼22,000. In this way we estimated that there would be ∼22,000 PYR cells that contributed to the LFP signal. We note that this would be an upper bound, as we assumed correlated activity across PYR cells and homogeneous extracellular electrical properties.

## Discussion

To a large extent, understanding brain function and coding requires that we are able to understand how oscillatory LFP signals are generated (Einevoll et al., 2013; Friston et al., 2015; Hyafil et al., 2015). Cross-frequency coupling analyses of LFP signals has led to ideas underlying learning and memory functioning (Canolty and Knight, 2010), and it is always important to do careful analyses (Scheffer-Teixeira and Tort, 2016). Further, given that particular inhibitory cell populations and abnormalities in θ rhythms are associated with disease states (Colgin, 2016), we need to consider how different cell types and pathways contribute to LFP recordings. Ultimately, the challenge is to bring together LFP studies from experimental, modeling and analysis perspectives. In this work, we make steps toward this challenge by gaining insight into the contribution of OLM cells to intrinsic θ rhythms as exhibited by an *in vitro* whole hippocampus preparation.

### θ rhythms and summary of results

The existence of θ rhythms (3–12 Hz) in the hippocampus has long been known, and these prevalent rhythms are associated with memory processing and spatial navigation (Colgin, 2013, 2016). These rhythms are present when the animal is actively exploring and during REM sleep. Further, they can be separated into higher or lower frequencies that are atropine resistant or atropine sensitive, respectively (Buzsáki, 2002; Colgin, 2013, 2016). Recent work has shown that low θ rhythms were elicited in rats with fearful stimuli and high θ with social stimuli (Tendler and Wagner, 2015). *In vitro* models of θ rhythms in the hippocampus have been developed (Gillies et al., 2002) as well as network mathematical models (Neymotin et al., 2011; Hummos and Nair, 2017), but it is challenging to bring about a mechanistic understanding of θ rhythms *in vivo* due to their various forms and pharmacological sensitivities combined with the interactions that occur between the hippocampus and other brain structures.

While it is clear that different interneuron subtypes are involved in θ rhythms (Colgin, 2013, 2016), it is difficult to untangle the cellular contributions to resulting θ rhythms exhibited in extracellular LFP recordings. That the required circuitry for θ rhythms has been shown to be present in local circuits of the hippocampus (Colgin and Moser, 2009) is both useful and helpful as it becomes more likely that biophysical LFP models can be linked to a cellular-based circuit understanding of θ rhythms. We took advantage of the *in vitro* whole hippocampus preparation that spontaneously expressed intrinsic θ rhythms (Goutagny et al., 2009), and previous inhibitory network models developed for this experimental context (Ferguson et al., 2015), to build biophysical LFP models.

The LFP is generated on the basis of transmembrane currents. This means that the LFP is a weighted sum of inward and outward currents. How the LFP changes as a function of location is not trivial. In our work here, when the LFP is governed by synaptic inputs the LFP peaks are narrower since the synaptic inputs are synchronized because of the coherent inhibitory spike rasters. On the other hand, LFP signals governed by return currents would produce LFP peaks that are less narrow as the signal slows down as it travels down the dendrites producing a time lag. This all thus translates to synaptic input location dependencies. Thus, while we can visualize and appreciate the synergistic balances between excitation and inhibition from different cell populations, we note that these combinations are not easily seen as summated balances. Signal decompositions and intuitions from many simulations are required. We leveraged our LFP models to make direct comparison with experimental LFP characteristics. This allowed us to constrain coupling parameters which in turn led us to understand the cellular contribution of interneuron subtypes, specifically OLM cells, to intrinsic θ LFP rhythms.

We showed how the extracellular θ field recorded along the cellular axis of a PYR cell was affected by the magnitude of the inhibitory synaptic currents inserted along its dendritic arbor. Fluctuations in the magnitude of the total inhibitory input occurred due to alterations in synaptic strength balances of the inhibitory networks. Our models exhibited network states in which interactions between OLM cells and BiCs could invert the polarity of the recorded signal and produce extracellular potentials of high or low magnitude. We also distinguished regimes where these cellular interactions preserved the frequency of the signal versus those that led to lags or abolishment of the extracellular LFP rhythm. When we applied experimental characteristics of θ frequencies and polarities to our biophysical LFP models, a clear selection emerged and thus we were able to constrain parameter values regarding connectivities. Specifically, we found that the connection probability from OLM cells to BiCs needed to be 0.21 and that synaptic conductances from OLM cells to BiCs had to be larger than 3.5 nS, and we called this the predicted regime.

Unexpectedly, we found that this predicted regime also exhibited a robust power output. That is, so long as parameter values were within the predicted regime, the power did not change (Fig. 7*A*), and in this regime we saw that BiCs were mostly silenced, BC/ACCs were significantly active while OLM cell activity decreased from high to low values as *g_bs_* increased (Fig. 2*C*). By decomposing the signal, we revealed that OLM cell inputs minimally contributed to the LFP power unlike the other cell populations (BiCs and BC/AACs or PV cells). The power of the OLM cell LFP component on its own, although low, showed some variation in the predicted regime [coefficient of variation (CV) < 1]. On the other hand, the power of the PV cell LFP component was a couple of orders of magnitude higher and showed more variation (CV > 1) in the predicted regime. This indicates that OLM cells contributed to LFP power robustness without contributing to average power whereas PV cells contributed to average power but their effect was more sensitive to perturbations in OLM-BiC interactions. Therefore, their contribution was variable. It is however interesting to note that the PV LFP component average power was larger than the average power of the predicted regime with all cells being present. Thus, our results indicated that adding OLM cells in the network can overall cause a small decrease in LFP average power as compared to when only PV cells were present and of course induce robustness. It was also interesting to observe that in almost half of the cases the OLM cell LFP component was arrhythmic or non-oscillatory despite the fact that OLM cells were driven by θ-paced EPSCs. That is, OLM cell inputs alone in most cases were not able to generate a θ LFP signal as recorded in the extracellular space of the PYR cell although OLM cell populations themselves were firing at θ frequency. Further LFP signal analysis decomposition showed that removing only basal excitation disrupted the robustness of the predicted regime. This suggests that a synergy of OLM cell inputs and basal excitatory inputs as coactivation of distal inhibition and proximal excitation is important to produce robustness in the predicted regime. Overall, an essential aspect in comparing model and experiment LFPs to predict model parameters and decipher cellular contributions was to match sources and sinks at different layers. Thus, having recordings from multiple layers is important.

### Morphologic details, synaptic locations, and related studies

As the main contribution to the LFP is thought to stem from synaptic input to neurons and the subthreshold dendritic processing, various studies have investigated how morphologic characteristics and intrinsic resonances shape the features of the LFP signal. In most cases input synapses are activated according to Poissonian statistics (Lindén et al., 2010; Łęski et al., 2013; Ness et al., 2016). However, in our study here, the origin population consisted of point neuron cell representations that had been constrained based on experimental patch clamp recordings from the whole hippocampus preparation. We used a scheme which is a combination of point neuron origin populations and a multi-compartment PYR cell model which served as a processor of synaptic inputs and produced the LFP. This scheme is conceptually very similar to the hybrid scheme proposed in Hagen et al. (2016).

One factor modulating the amplitude of LFPs was related to the somatodendritic location of synaptic inputs on the PYR cell tree. Different populations of GABAergic interneurons target different dendritic domains and the domain-specific targeting of various interneurons supports the hypothesis of domain-specific synaptic integration in CA1 PYR cells (Spruston, 2008). In CA1 PYR cells, distal and middle apical dendrites comprise two distinct dendritic domains with separate branching connected by a thick apical dendrite. This cytoarchitectonic separation of the cluster of distal dendrites relative to middle and proximal dendrites was shown to critically reduce the effect of distal EPSCs to somatic excitability (Srinivas et al., 2017). The presence of a single apical dendrite with many obliques in stratum radiatum caused a large shunting of EPSCs traveling from the tuft dendrites to the soma. Thus, we can appreciate our observation that OLM cells, which target distal dendrites, minimally affected LFP power in SP considering the limited ability of distal inhibition to reach more proximal and somatic regions of the CA1 PYR where maximum power was recorded. This is not just due to the distal location of these inputs but more due to the cytoarchitectonic separation of the cluster of distal dendrites relative to middle and proximal dendrites. This separation prohibited inhibitory inputs in distal regions from effectively propagating to somatic and proximal regions of CA1 PYR cells and thus being reflected in the extracellular space.

We can further consider our results in light of another theoretical modeling study by Gidon and Segev (2012), which showed that inhibitory inputs can affect excitatory inputs locally and/or globally, depending on the relative locations of the excitatory and inhibitory synapses. In particular, this can help us understand the loss of robust power in the predicted regime after removal of OLM cells. The predicted regime consists of different connectivities that generated different spiking patterns that gave rise to fluctuations in inhibitory input in different synaptic locations. First, inhibitory input hyperpolarized the membrane potential, which resulted in shunting of the adjacent dendritic compartments. Activation of excitatory synapses within the shunted compartments will thus generate smaller depolarization, compared with non-shunted dendrites (“local” effect). Second, the local shunting would suppress excitatory input in a nonlinear fashion at locations that were not directly affected by the shunting (“global” effect). Thus, when inhibitory inputs were activated simultaneously with excitatory inputs, the average (i.e., across trials) evoked membrane potential within shunted dendritic compartments should be smaller compared with compartments that had no inhibitory input. At the same time, excitatory effects throughout the entire dendritic tree would be reduced in a nonlinear fashion, and which can be quantified as the change (with vs without inhibitory input) of the trial-to-trial variability of the membrane potential. In our case the activation of excitatory inputs occurred in regions not close by the OLM cell inhibitory inputs, thus the overall power did not increase but the robustness was affected. In Gidon and Segev (2012), the authors examined the spread of shunt level implications using a CA1 reconstructed neuron model receiving inhibition at three distinct dendritic subdomains: the basal, the apical, and the oblique dendrites as innervated by inhibitory synapses. They found that the shunt level spread effectively hundreds of micrometers centripetally to the contact sites themselves spanning from the distal dendrites to the somatic area. This observation thus showed that the somatic area was indeed influenced by shunting inhibition which means that excitatory input non-linearities in our model will be reduced in the presence of global inhibition in the somatic area leading to a decrease in variability and thus robustness in the membrane potential. Of course, the LFP is a measurement of transmembrane currents and not membrane potential. However, the reduction of excitatory input mediated non-linearities will also reduce the variability in the distribution of return currents and thus the variability in the LFP.

### Limitations and future considerations

Our present study was limited in terms of not considering more inhibitory cell types (Bezaire et al., 2016) and by considering ongoing intrinsic θ rhythms since θ frequency inputs were used (Fig. 1). However, our inhibitory network models were constrained by the experimental context and our less complex model representations enabled us to explore many thousands of simulations and directly compare our biophysical LFPs with experimental LFP features. This aspect was key in allowing us to constrain parameter value sets and to gain insights.

θ rhythms are foremost generated due to subthreshold activity and dendritic processing of synaptic inputs. Here, we used a passive PYR cell model as the spiking component has been shown to mainly contribute to the LFP at frequencies higher than 90 Hz (Schomburg et al., 2012), while the active voltage-gated channels that were eliminated here were shown to influence LFP characteristics more prominently in frequencies above the θ range (Reimann et al., 2013). Thus, although the presence of voltage-gated channels will influence the exact distribution of return currents, we thought that it was a reasonable simplification to not include them in this study. Indeed, in an additional set of simulation (data not shown), we observed that the presence of hyperpolarization-activated cyclic nucleotide-gated (HCN) channels on the PYR cell did not influence the sink-source LFP profile and frequency examined here, although it did affect the wave form characteristics.

Another limitation is the usage of a single PYR cell to predict network dynamics. However, we note here that since the LFP is a linear summation of the transmembrane currents in the extracellular space (Equation 1), incorporating more PYR cells could result in a linear additive effect in the extracellular space. This would lead to the same LFP profiles as in the case of a single cell only significantly magnified provided that the cells have a similar morphology, physically arborizing in ways that facilitate superposition rather than cancellations of fields, and receive similar presynaptic inputs. Indeed, there is a homogeneous cytoarchitecture disposition of the PYR cells across the CA1 layer (Andersen et al., 2006) and is one of the factors responsible for the extracellular sinks and sources recorded in CA1. Also, PYR cells receive similar presynaptic inputs from the presynaptic populations which project on the same layers across cells. For this reason, we do think that the conclusions derived from the single cell LFP output will remain on the network level to some extent. Of course, important variabilities across PYR cells also exist and considering them in future studies will be important (Soltesz and Losonczy, 2018). Therefore, careful network modeling will be required to assess the network-generated LFP output.

Extracellular studies suggest that the main current generators of field θ waves are the coherent dendritic and somatic membrane potential fluctuations of the orderly aligned PYR cells (Winson, 1978; Buzsàki and Eidelberg, 1983; Brankačk et al., 1993). Thus, distal and local ascending pathways onto PYR cells can in principle contribute to extracellular LFP deflections. To understand θ rhythms one needs to consider the populations projecting onto the PYR cells in CA1. During *in vivo* behaviors, medial septum and entorhinal cortical inputs onto CA1 PYR cells are prominent modulators of the amplitude, phase and wave form features of θ rhythms in conjunction with local inhibitory and excitatory cells. However, spatiotemporal coincidence of inputs makes separation difficult and thus it is challenging to determine cellular contributions to LFP recordings. As there is significant spatiotemporal overlap on PYR cell dendrites across ascending pathways it would be hard to disentangle the cellular composition of these pathways and assess the cellular contribution to θ LFP characteristics. As shown in previous studies (Makarova et al., 2011) blind separation techniques such as Independent Component Analysis produce poor results when trying to disentangle combinations of rhythmic synaptic sources with extensive spatiotemporal overlap. By focusing on intrinsic θ rhythms in the *in vitro* whole hippocampus preparation here, we reduced the spatiotemporal overlap of different pathways and unraveled the cellular composition of the different pathways projecting to the PYR cell. We were thus able to decipher the contribution of OLM cells to intrinsic θ rhythms. This work could potentially be used as a basis to understand OLM cell contributions during *in vivo* θ LFP recordings.

Moving forward we aim to take advantage of the insights gained here to build hypothesis-driven θ generating networks. In this way, we hope to be able to determine the contribution of different cell types and pathways to LFP recordings that are so heavily used and interpreted in neuroscience today.

10.1523/ENEURO.0146-18.2018.supp1 Supplementary latex zip. Download supplement 1, ZIP file

10.1523/ENEURO.0146-18.2018.supp2 Supplementary Code. Download supplement 2, GZ file

## Synthesis

Reviewing Editor: William Stacey, University of Michigan Decisions are customarily a result of the Reviewing Editor and the peer reviewers coming together and discussing their recommendations until a consensus is reached. When revisions are invited, a fact-based synthesis statement explaining their decision and outlining what is needed to prepare a revision will be listed below. The following reviewer(s) agreed to reveal their identity: Gaute Einevoll, Iris Oren.

The reviewers and I have discussed your paper and find it of potential interest. There are several questions that we had that should be addressed as a revision.

1. Address the concern of Reviewer 1 about simulating the LFP from just one pyramidal cell.

2. Address the first concern of Rev 2 about the structure of the model. This is the main concern we have. The issue is that the model shows that the connections that you modeled are sufficient for generating theta. However, we are concerned that at the implicit conclusion that the parameter values identified are “true”. As we see it, your theta rhythmicity of interneurons is generated by (A) the excitatory input to the network (B) the int-int connections. This argues that PC-Int connections are not necessary for generating theta in a network with this connectivity. However, a previous model of CA3 theta showed that PC-OLM and PC-BC connection strengths were important in generating theta rhythmic firing of PCs when rhythmic input was absent (Hummos & Nair, PLoS One, 2017). There is also past work in this field, such as Tort et al PNAS 2007, 2009 showing that those connections might be important. Although the model differs in several features, thus there is some prior evidence that PC-interneuron reciprocal connection would alter the features of the theta parameter space. Thus, the emphasis of the uniqueness of the parameters identified in the present work needs to be toned down, and this limitation should be discussed thoroughly.

3. Clarify how the theta frequency was evaluated (Item 2 from Rev 2). Also please answer the questions about relative power (Item 4, R2). In this latter case, using power ratios is a well known method as the reviewer describes.

4. Clarify and show how the interneurons' theta rhythmicity affected the simulation (Item 3, R2)

5. Item 5 from R2 suggests including schematics. You do not necessarily have to have a schematic for every iteration, but clearly it was difficult to follow. Please consider adding several schematics to make it more readable.

6. The code was not accessible to the reviewers because they did not have sufficient software. Please assure the code is accessible to all.

7. The minor points from both reviewers should be addressed.

Reviewer 1:

The paper addresses an important question, namely the use of local field potentials (LFPs) in neural circuits to constrain neural network models. They combine (i) a previous model for hippocampal network activity derived by their group with (ii) a biophysical modeling scheme for LFPs to (iii) model experimental data for in vitro preparations of the same system recorded from their own group. In particular, they systematically explore what model parameters makes their model prediction be in accordance with their experimental data, thereby gaining new understanding of the system. I find the approach to be sound and the work very accomplished. The paper is also well written. Since I also find the questions addressed to be both important and timely, I am supportive of publishing of this work in eNeuro.

I have however some points that must be addressed:

1. It seems like in the forward modeling of the LFP only a single pyramidal neuron is assumed to generate the LFP. In practice, I assume that several pyramidal neurons contribute to the LFP recorded in the in vitro preparations. Is it a good approximation to assume only a single LFP-generating neuron? By the way: LFPy allows for modelling of LFPs from a population of neurons (see, e.g., example 3 in the LFPy-article by Linden et al, Frontiers in Neuroinformatics, 2014).

Minor points:

* l. 688: It is stated that the connection probability between OLM cells and BiCs is 0.21. How precise is this number? I assume you mean a range around 0.21?

* l. 707: electron-centric -> electrode-centric

* l. 709: recored -> recorded

* l. 769: You write: “That is, so long as parameter values were within the predicted regime, the power did not change (Fig 6A), and in this regime we see that BiCs are mostly silenced, BC/ACCs are significantly active while OLM cell activity decreases from high to low values as gbs increases (Fig 2C). By decomposing the signal we revealed that OLM cell inputs minimally contributed to the LFP power unlike the other cell populations (BiCs and BC/AACs or PV cells).” Seems strange that BiCs are mostly silenced, yet contribute to the LFP power.

Reviewer 2

The authors have utilized a previously developed conductance model of hippocampal CA1. Where possible, connection probabilities and synaptic parameters have been based on experimental findings. The present study presents a systematic exploration of parameter space regarding the connections between bistratified and OLM cells (c_sb, g_bs, g_sb), and the consequences for theta frequency oscillogenesis. The present study builds on previously published models in two main ways: 1) by implementing a biophysical computation of the LFP resulting from the membrane currents. 2) by including recurrent excitatory input onto CA1 basal dendrites.

The authors first show that in a model without basal excitation, the characteristics of the LFP are sensitive to g_sb and g_bs., and, as expected, firing rates of the different cell types also vary. They then include basal excitation in the model, and show that certain regions of parameter space generate oscillations with physiological reversal profiles and frequency. They use these as criteria for defining a subset of parameter values that are robust for oscillogenesis. They study this parameter region for effects of removing OLM or PV cells, and basal excitation from the network. They show that OLM-Pyr connections generate the robust parameter space, while removal of PV-Pyr connections reduces the peak LFP power, and removal of basal excitation reduces the peak power. Lastly, they use their biophysical model to estimate the number of cells contributing to the LFP signal.

The report presents a powerful tool for studying the contributions of the activity of specified cell types and connections for the generation of LFP signals. While I have concerns about the implementation of the model and conclusions drawn (see below), the framework that they have developed will provide a basis for exploring more physiological models in future.

Main concerns:

1) The model lacks connections which are likely important for theta frequency oscillogenesis. Namely, the Pyr - interneuron recurrent connections. The exploration of parameter space and the selection of values (c_sb=0.21) is sensitive to network structure. Hence, it is possible that adding these (or other) connections will alter the conclusions. This should be discussed.

2) It is not clear how the LFP theta oscillations for each simulation were assessed. In evaluating the rejected and accepted parameters (Fig 4- 6), was the criterion that the power spectrum needed to show a peak in the theta frequency range. For Fig 7-11, the power of the peak frequency is used, but it is not apparent if this peak was always in the theta band for each simulation. How was the LFP peak frequency assessed for each simulation, and when determining the peak power in Fig 7-11, was there a criterion that the peak frequency needed to be in the theta band? This information needs to be included. The conclusions of the role of the O-LM and PV cell contributions to the LFP are dependent on this. For example, a situation in which the LFP shows white noise could also have a high peak LFP power, but is not comparable to the theta peak LFP power in the predicted regime. Exemplary power spectra for all panels in Fig 7 should also be added.

3) The argument being put forward is that “cancellations and rhythm loss occurs due to interactions between OLM cells' synaptic and return currents and excitatory inputs”. An additional factor that could account for the loss of rhythmicity is the loss of theta frequency activity of the interneurons. Was the theta-frequency firing of interneurons validated for each simulation? The theta rhythmicity of inhibition should be reported.

4) Similarly, assessing the relative power in theta vs other frequencies would be informative. For example, the present analysis does not distinguish between irregular LFP, and a reduced theta power (eg. as exemplified in the three panels of Fig9B). Calculating the ratio of theta to a different band (e.g. delta) could be informative.

5) On the whole, the writing is very dense. It would be helpful if schematics are included in diagrams wherever possible. These should show interneuron activity (high/low rate and rhythmicity) and the dominant currents. For example, in Fig 9, the 6 panels would be much easier to understand if there was a visual schematic for each.

Minor points:

1) The choice of abbreviation g_sb and g_bs is not evident. Perhaps change to g_ob/g_bo?

2) The abbreviations PV and SOM need to be stated in first instance in the methods (line 112)

3) Line 117: It is not clear what is meant by peak alignments. Are these alignments to the phase of the theta oscillation?

4) Line 188 state that spike rasters for BiC wre used to model excitatory input. What was the motivation for the selection of these specific BiC rasters? Please also specify c_sb.

5) Lines 209 - 218. This discussion of results is out of place in the methods section and difficult to understand.

6) Lines 242, repetition of 142-143

7) Figures 1, 2A, 10, 11 should use labeled sub-panels. It is difficult to follow with references to “top”, “bottom”

8) Figure 2A: It is confusing that the dark is above the light for all the rasters, but that it is inverted for the LFP trace.

9) The text from lines 414-442 is very difficult to follow. The “four regions” in the 2A colour plot are not evident. Addition of schematics in the figure to accompany the text would be helpful.

10) Lines 461-470 set out various predictions for the full model, but these are not referred to again. It would be helpful for the reader if each of these predictions are addressed explicitly and revisited.

11) What was the upper band for theta frequency used

12) Figure 5 legend should be changed to “...parameter sets using different LOWER frequency bounds”

13) Lines 558-563: I do not understand the argument being put forward about the OLM cell LFP component. Please rephrase

14) Line 585-586: “It is clear from the magnitude of the signal power that it is indeed mainly due to the component from PV cells”. The reasoning in this sentence seems awry. The signal is due to the PV cells, because that is how the network has been set up. But this is not seen from the magnitude of the signal power.

15) Legend to Fig 7: Please specify that the plots show peak power at electrode of maximal LFP theta power.

16) Figure 7A: Inset is too small

17) Line 584: Why are you considering g_bs=0 for mimicking OLM optogenetic silencing? The bistratified synapses would still be functional in this situation

18) Lines 586: “Consider that for the parameter regime g_sb=0 ...” is this referring to the intact model in 7A, or the PV only network of 7C? Please specify.

19) For the text accompanying Fig 9, it would be helpful to state explicitly what you are trying to show in this figure. Why were the particular examples selected?

20) Lines 639-644:Figure 9 does not reveal that that the contribution of OLM cells is more dependent on g_bs due to the colour scaling. This is only apparent in Fig 10, middle, left. These lines seem to be saying the same thing three times. Please make this more concise.

21) Line 657-666: These are discussion points.

## Author Response

Point-by-point reply to the reviews

Synthesis of Reviews:

Computational Neuroscience Model Code Accessibility Comments for Author (Required): There are concerns that the code could not be tested as presented because software was missing. Please assure the code is accessible to all.

We apologize that the code was not easily available to the reviewers. We have now made changes to the repository to make the code easier to access and run.

Please note the following: 1. Before performing any simulations, ensure to install the following packages: Python, LFPy and NEURON. Links for installation are provided in the code documentation. 2. To perform simulations, users are required to download spike times from OSF (osf.io/vw3jh) for the connection probability of choice. This is likely the step that was not clearly explained before and confused the reviewers.

We have now made the above steps clearer in the code documentation. We have also included the spike times for the connection probability csb=0.21 so that a full connection probability example is available. The users would need to download the spike times for the rest of the connection probabilities if they wish to reproduce results for those.

We have also organized the code according to the sequence of the Figures that appear in the manuscript.

Significance Statement Comments for Author (Required): N/A

Comments on the Visual Abstract for Author (Required): N/A

Synthesis Statement for Author (Required): The reviewers and I have discussed your paper and find it of potential interest. There are several questions that we had that should be addressed as a revision.

1. Address the concern of Reviewer 1 about simulating the LFP from just one pyramidal cell.

A detailed response is provided below in responding to Reviewer 1.

2. Address the first concern of Rev 2 about the structure of the model. This is the main concern we have. The issue is that the model shows that the connections that you modeled are sufficient for generating theta. However, we are concerned that at the implicit conclusion that the parameter values identified are “true”. As we see it, your theta rhythmicity of interneurons is generated by (A) the excitatory input to the network (B) the int-int connections. This argues that PC-Int connections are not necessary for generating theta in a network with this connectivity. However, a previous model of CA3 theta showed that PC-OLM and PC-BC connection strengths were important in generating theta rhythmic firing of PCs when rhythmic input was absent (Hummos & Nair, PLoS One, 2017). There is also past work in this field, such

2

as Tort et al PNAS 2007, 2009 showing that those connections might be important. Although the model differs in several features, thus there is some prior evidence that PC-interneuron reciprocal connection would alter the features of the theta parameter space. Thus, the emphasis of the uniqueness of the parameters identified in the present work needs to be toned down, and this limitation should be discussed thoroughly.

Thank you for pointing out this concern about the structure of the model. We agree with the reviewers that PC-Int connections are important for theta rhythm generation. However, we note that we are not claiming that the PC-Int connections are not necessary for generating theta rhythms. Rather, in our structure the rhythmicity of the interneuron populations is directly imposed via the applied input EPSCs. Thus, the theta rhythmicity is imposed on the inhibitory network and is not generated via PC-Int interactions. We explain in detail below.

There are two essential aspects to bring forward to address these concerns.

First, the LFP modeling study in this work is not about the generation of theta rhythms directly and specifically. Rather, it is about developing biophysical LFP models that build on network modeling work done in Ferguson et al. (2015) to allow comparison with experimental LFPs etc. In Ferguson et al., a focus (also in the present work) was in regards to how OLM and BiC interactions affect theta power, where the theta rhythm was based on an ongoing intrinsic theta rhythm present in a whole hippocampus preparation (Goutagny et al. 2009). Our representation of the ongoing theta meant that we inserted theta-frequency inputs, EPSCs directly based on experimental measurements from the whole hippocampus preparation, into the inhibitory cell model populations. The cellular models were directly based on the whole hippocampus preparation. As such, Ferguson et al. (2015) was not intended to specifically address the generation of theta rhythms per se, but was able to make suggestions/predictions about the power of the ongoing theta rhythm. This is noted at the beginning of the 2nd paragraph of the ‘Materials and Methods’ section of Ferguson et al. (2015): “We note that our focus is on the power, and not on the frequency, of theta oscillations. This allows us to utilize actual excitatory postsynaptic current (EPSC) traces, recorded from putative OLM and PV+ interneurons under voltage clamp in the intact hippocampus in vitro, to drive our individual interneurons. In this way, we can simplify our network interactions so that they do not include feedback excitation from PYRs. This simplification is important, as PYR networks on their own may produce complex spiking and bursting behaviors, and thus make the microcircuit too complex (i.e., far too large of a parameter space) to be able to understand how BiC-OLM cell interactions affect network rhythms.” And following the summarizing results in Discussion of Ferguson et al. (2015), it is noted in the 2nd paragraph of the Discussion that: “These complex regimes are created in a highly non-linear manner by the balances between OLM and BiCs. This balance affects the precise timing of the various cell populations, in turn affecting how each population influences network activity”. And, as noted in the present manuscript referring to results from Ferguson et al. (2015), lines 318-322: “OLM cells could play a small or large role in the resulting theta power depending.” It was also noted in the Discussion of Ferguson et al. (2015) that: “Given our simplistic LFP representation, it is important to incorporate biophysically realistic models of the LFP to represent our network activity (e.g., Einevoll et al., 2013), with the aim of facilitating the comparison between our model and experimental network theta rhythms.” This is what we have done in the present work, and developing, using and comparing our biophysical LFP models with experiment al LFPs, we have gained insight as detailed in the present paper.

In summary, we agree with the reviewers that if the main focus is on theta rhythm generation per se, it is important to directly include feedback excitation to inhibitory cell populations, as has been done in the cited studies, and most recently in a detailed CA1 microcircuit model of Bezaire et al. (eLife 2016) with biophysical LFP models. Indeed, we have done other network modeling to examine theta rhythm

3

generation directly, taking advantage of the whole hippocampus preparation and previous theoretical insights etc. (Ferguson et al. eNeuro 2017), but not (yet) with the inclusion of biophysical LFP models.

The general strategy in the present work (and in Ferguson et al. 2015, 2017) is to be able to do thorough parameter explorations to address particular questions and in which a biological (interactive) link was possible, so reducing the network complexity and focusing the questions where possible was a key aspect. Indeed, as noted by Reviewer 2, we hope that this work will “provide a basis for exploring more physiological models in future.” Therefore, any parameter values identified as “true” are necessarily in the context of the model exploration question (i.e., OLM and BiC interactions) and experimental estimates.

As such, we believe that our work here has uncovered a potentially important robust theta aspect regarding distal inhibition and basal excitation, which likely would've been difficult if not impossible to extract if a full-scale microcircuit model had been used at the beginning.

Second, decoupling a point neuron network model and multi-compartment neuron model for the LFP generation has been done previously for cortical circuits, in a so-called hybrid approach (Hagen et al. Cerebral Cortex 2016). Our work here can be seen as such a decoupling with the point neuron network model of Ferguson et al. (2015) being the ‘generator’ of the LFP signal and the PYR multi-compartment model the ‘processor’ of the LFP as noted on lines 348+. It was important that our microcircuit models were constrained in ways (size, numbers, cellular properties, EPSC theta-frequency inputs etc.) representative of the whole hippocampus preparation. In this way, we were able to do direct comparisons between model and experiment LFPs and gain some insights to use and translate in full-scale systems of large networks of multi-compartment models with biophysical LFP models in our future studies.

We have added a few sentences (lines 324-329 in revised ms) just before the Results section (‘Overall characteristics of biophysical LFP models’) to clarify our model structure and address this concern.

3. Clarify how the theta frequency was evaluated (Item 2 from Rev 2). Also please answer the questions about relative power (Item 4, R2). In this latter case, using power ratios is a well known method as the reviewer describes.

A detailed response is provided below in responding to Reviewer 2. In essence, we did a peak detection to obtain frequencies, and although computing power ratios is potentially helpful, the EPSCs that drive the network are specifically in the theta range. Thus our model structure was specific for ongoing theta rhythms and so looking at the emergence of other frequencies is unlikely to be helpful in the present situation.

We note here that other than the imposed theta frequency (of approx. 5.8 Hz) the network could only exhibit “loss of peaks” in the extracellular space mainly due to synaptic-return current cancellations. This loss of peaks did not necessarily translate into the emergence of a slower regular rhythm but rather in an irregular and arrhythmic LFP. This is why we refrained from characterizing the slower LFP traces resulting from peak loss as slower frequency bands.

4. Clarify and show how the interneurons' theta rhythmicity affected the simulation (Item 3, R2)

A detailed response to this and other points raised by Reviewer 2 is provided below. We think that this point, the above point here, and other ones raised by Reviewer 2 below relate to the structure of our

4

model (point 2 above). We have provided a detailed response to point 2 above, and as mentioned, we have revised our manuscript to make this clear.

5. Item 5 from R2 suggests including schematics. You do not necessarily have to have a schematic for every iteration, but clearly it was difficult to follow. Please consider adding several schematics to make it more readable.

Thank you for the suggestion. We have added schematics as suggested -- specifically, to Figures 9 and 10.

6. The code was not accessible to the reviewers because they did not have sufficient software. Please assure the code is accessible to all.

Please see our response above in the Synthesis of reviews.

7. The minor points from both reviewers should be addressed.

We have addressed all minor points. Please see specific responses to both Reviewers.

We would like to thank the reviewers for their detailed comments that have helped us present our work more clearly. We have responded to the synthesis points above and further details (as well as referring to the synthesis points where detail was already provided) are provided in the point-by-point responses below.

We would also like to point out that we revised our whole manuscript for tense consistency, and converted to using past tense for work done/observed and present tense when expressing our conclusions/observations, as is more typically done in the literature.

Reviewer 1:

The paper addresses an important question, namely the use of local field potentials (LFPs) in neural circuits to constrain neural network models. They combine (i) a previous model for hippocampal network activity derived by their group with (ii) a biophysical modeling scheme for LFPs to (iii) model experimental data for in vitro preparations of the same system recorded from their own group. In particular, they systematically explore what model parameters makes their model prediction be in accordance with their experimental data, thereby gaining new understanding of the system. I find the approach to be sound and the work very accomplished. The paper is also well written. Since I also find the questions addressed to be both important and timely, I am supportive of publishing of this work in eNeuro.

We thank the reviewer for his/her appreciation of our work.

I have however some points that must be addressed: 1. It seems like in the forward modeling of the LFP only a single pyramidal neuron is assumed to generate the LFP. In practice, I assume that several pyramidal neurons contribute to the LFP recorded in the in vitro preparations. Is it a good approximation to assume only a single LFP-generating neuron? By

5

the way: LFPy allows for modelling of LFPs from a population of neurons (see, e.g., example 3 in the LFPy-article by Linden et al, Frontiers in Neuroinformatics, 2014).

The reviewer is correct that a single pyramidal neuron was used in computing our biophysical LFP, and also in assuming that several pyramidal neurons would contribute to the LFP recording in the in vitro preparations. Also, we thank him/her for pointing out the example 3 regarding MPI.

In the present study, we examined the extracellular potential output of a single cell which we referred to as the LFP. This was mainly for consistency with the computational literature (e.g., Linden et al 2010, Leski et al 2013 etc.) where the term LFP has been used referring to the extracellular field from single or multiple cells.

Besides terminology, the LFP would be generated as a result of network activity and whether and how our results would translate to the network level remains to be fully examined. However, we do think that the conclusions derived from the single cell LFP output will remain on the network level to some extent for the following reasoning. The LFP is a linear summation of the transmembrane currents in the extracellular space, therefore it can be easily deduced that incorporating more cellular units that produce the same fields in the context of a network will result in a linear additive effect in the extracellular space. This would lead to the same LFP profiles as in the case of a single cell only significantly magnified (we directly illustrate this below in the figure below where 20 pyramidal cells are used). For such an effect to occur it is required that all cellular units produce the same laminar LFP profiles and for this to occur several conditions need to apply. Cells need to be similar in morphology, and they need to be physically arborized in a way that facilitates superposition of fields rather than cancellations. Also, the presynaptic inputs, EPSCs or IPSCs, need to be similar across cells. Pyramidal cells in the CA1 share many similarities. There is a homogeneous cytoarchitecture disposition of the pyramidal cells across the CA1 layers with their somata located in the stratum pyramidale, the basal dendrites in stratum oriens and the apical and tuft dendrites in stratum lacunosum moleculare. This uniform arborization of neurons is also one of the factors responsible for the large extracellular sinks and sources recorded in CA1. Also, pyramidal cells possibly receive similar presynaptic inputs from the presynaptic populations projected upon the same layers across cells. However, important variabilities across pyramidal cells also exist and considering them will be important (e.g., see Soltesz and Losonczy, Nature Neuroscience 2018 review). Since slow oscillations such as theta are mainly generated by synaptic events instead of extracellular action potentials (Schomburg et al. 2012), it is reasonable to assume that the single cell LFP output will be relevant in the context of the network. However, careful network modelling will be required to assess the network-generated LFP output.

We have expanded the section in the Methods (‘Biophysical computation of LFP’) to make it clear that our use of LFP is for terminology reasons and that if cell units can be assumed to be similar, there would be a linear summation for many cell units in the resulting LFP.

The figure (as referred to above) shows the LFP generated by 20 cells. The signals generated by the various cells contribute in a distance dependent fashion to the recording electrode according to the equation below. Subsequently, every one of these distance-scaled contributions add up linearly to give rise to the total LFP.

6

Minor points: * l. 688: It is stated that the connection probability between OLM cells and BiCs is 0.21. How precise is this number? I assume you mean a range around 0.21? This number is not intended to be precise, and connection probabilities explored were estimated from the literature (see section 2.3.3 in Ferguson et al. 2015). However, within the range of explored probabilities, the work here suggests that 0.21 would be an appropriate connection probability to allow the resulting model LFP to represent LFPs from experiment (Figure 4).

* l. 707: electron-centric -&gt; electrode-centric Thank you, corrected in the manuscript.

* l. 709: recored -&gt; recorded Thank you, corrected in the manuscript.

* l. 769: You write: “That is, so long as parameter values were within the predicted regime, the power did not change (Fig 6A), and in this regime we see that BiCs are mostly silenced, BC/ACCs are significantly active while OLM cell activity decreases from high to low values as gbs increases (Fig 2C). By decomposing the signal we revealed that OLM cell inputs minimally contributed to the LFP power unlike the other cell populations (BiCs and BC/AACs or PV cells).” Seems strange that BiCs are mostly silenced, yet contribute to the LFP power. We note that we did not separately decompose BiCs and BC/AACs in the PV cell grouping, mainly due to the experimental data only distinguishing between SOM and PV cell types. Thus, the exact contribution of BiCs versus BC/AACs in this regime is not specified. However, given our results (Fig 7A-C), it is

7

likely that the BC/AACs are responsible for the high power in this predicted regime. We also note that the normalization in Fig 2C is for each cell type, and so BiCs are low (somewhat silenced) in this regime, they are not fully silenced. We have adjusted the wording in this identified part of the manuscript to include the above expanded reasoning.

Reviewer 2:

The authors have utilized a previously developed conductance model of hippocampal CA1. Where possible, connection probabilities and synaptic parameters have been based on experimental findings. The present study presents a systematic exploration of parameter space regarding the connections between bistratified and OLM cells (c_sb, g_bs, g_sb), and the consequences for theta frequency oscillogenesis. The present study builds on previously published models in two main ways: 1) by implementing a biophysical computation of the LFP resulting from the membrane currents. 2) by including recurrent excitatory input onto CA1 basal dendrites.

The authors first show that in a model without basal excitation, the characteristics of the LFP are sensitive to g_sb and g_bs., and, as expected, firing rates of the different cell types also vary. They then include basal excitation in the model, and show that certain regions of parameter space generate oscillations with physiological reversal profiles and frequency. They use these as criteria for defining a subset of parameter values that are robust for oscillogenesis. They study this parameter region for effects of removing OLM or PV cells, and basal excitation from the network. They show that OLM-Pyr connections generate the robust parameter space, while removal of PV-Pyr connections reduces the peak LFP power, and removal of basal excitation reduces the peak power. Lastly, they use their biophysical model to estimate the number of cells contributing to the LFP signal.

The report presents a powerful tool for studying the contributions of the activity of specified cell types and connections for the generation of LFP signals. While I have concerns about the implementation of the model and conclusions drawn (see below), the framework that they have developed will provide a basis for exploring more physiological models in future.

We thank the reviewer for his/her analysis and appreciation of the basis of our study.

Main concerns: 1) The model lacks connections which are likely important for theta frequency oscillogenesis. Namely, the Pyr - interneuron recurrent connections. The exploration of parameter space and the selection of values (c_sb=0.21) is sensitive to network structure. Hence, it is possible that adding these (or other) connections will alter the conclusions. This should be discussed.

We have provided a detailed response and discussion to this above (point 2 in the synthesis of reviews).

Overall, we note that in the study here we have focused on the generation of extracellular fields produced by the processing of synaptic inputs by pyramidal cells rather than the generation of the theta rhythms directly and specifically. As such, the modelling done here is conceptually different from what it would have been if we were modelling the emergence of theta.

We agree with the reviewer that if the focus was on theta frequency oscillogenesis, selection of c_sb values would be sensitive to network structure in this way. However, as noted above, the focus was on

8

the effect on extracellular LFP generation. That is, on the pyramidal cell and the inputs that it receives while the (ongoing) theta frequency is imposed on the model through the EPSCs that drive the interneurons. Even though we haven't explicitly modeled the connection between the pyramidal cell and the interneurons, the EPSCs imposed on the interneurons encompass the total amount of excitatory inputs that interneurons receive under in vitro experimental conditions which include inputs from other pyramidal cells. So, one can consider that these inputs are indirectly present.

As mentioned in summary above, we agree with the reviewers that if the main focus is on theta rhythm generation per se, it is important to directly include feedback excitation to inhibitory cell populations, as has been done in the cited studies, and most recently in a detailed CA1 microcircuit model of Bezaire et al. (eLife 2016) with biophysical LFP models. Indeed, within our own group too we have done other network modeling to examine/explain theta rhythm generation directly, taking advantage of the whole hippocampus preparation and previous theoretical studies . (Ferguson et al. eNeuro 2017), but not (yet) with the inclusion of biophysical LFP models.

2) It is not clear how the LFP theta oscillations for each simulation were assessed. In evaluating the rejected and accepted parameters (Fig 4- 6), was the criterion that the power spectrum needed to show a peak in the theta frequency range. For Fig 7-11, the power of the peak frequency is used, but it is not apparent if this peak was always in the theta band for each simulation. How was the LFP peak frequency assessed for each simulation, and when determining the peak power in Fig 7-11, was there a criterion that the peak frequency needed to be in the theta band? This information needs to be included. The conclusions of the role of the O-LM and PV cell contributions to the LFP are dependent on this. For example, a situation in which the LFP shows white noise could also have a high peak LFP power, but is not comparable to the theta peak LFP power in the predicted regime. Exemplary power spectra for all panels in Fig 7 should also be added.

We thank the reviewer for pointing this out. Indeed, we did not clearly specify how the LFP theta oscillations were assessed. Specifically, we applied a peak detection on the LFP trace and use a threshold to avoid baseline peaks. The threshold used is empirical to the data but typically was 0.0001 microV. We required that the number of peaks be larger than 15 which given the 5 sec LFP trace corresponds to 3 Hz (the lower bound used in Figure 4). We have added this information to the revised manuscript.

As noted on lines 490-492, in evaluating the rejected and accepted networks, the network was “accepted” if it was in theta frequency range across all layers (as well as the polarity profile requirement). Thus, the power spectrum would have a clear peak in the theta frequency range. This is specifically seen in the visualized example of Figure 7A. The selection of c_sb=0.21, and the ‘right part’ (Figure 6) gives the ‘accepted’ parameters. This means that there is definitely a theta frequency in these parameter sets. Due to the structure of our model, once the lower bound was imposed, the frequency cannot be greater than the ‘imposed’ (via EPSCs to inhibitory populations) theta frequency of about 6 Hz. (see lines 496+). Thus, moving forward from Figure 7-11, there is necessarily a theta frequency across layers for these parameters (when the network is intact). Thus, conclusions regarding OLM and PV cell contributions can be made in this light.

As noted on lines 542-546, we used the power (amplitude) of the theta peak (maximal one across the layers) as the color in Figure 7 (as determined via FFT spectral analysis), and which we show later in Figure 11 to be in the somatic regime. As mentioned above, this ‘accepted parameters’ regime necessarily has a theta frequency. However, it is possible that the ‘left part’ does not necessarily have a LFP theta frequency (i.e., not in the predicted regime). Regardless, it is clear that there is quite a variation in the maximal peak power across the layers, unlike the ‘right part’ in the accepted parameter regime.

9

This is also seen to be the case for Figure 7B-C and Figure 10 when parts of the network are removed. We have added a sentence in this part to ensure that this is clear.

As noted above, due to the nature of the model structure (ongoing theta rhythm) it is not possible to have an LFP frequency greater than the imposed 6 Hz one (i.e., LFP would not show white noise). In the predicted regime, the peak power is necessarily theta. However, once the decomposition is done to remove the contribution of the different cell types, the LFP theta frequency can be lost, and this happens in different ways across the layers as shown in Figure 9. Rather than show exemplary power spectra for all the panels in Figure 7, we have shown the various ways in which the LFP theta is lost in Figure 9. We thought that this would be more useful than the power spectra on its own which would not show the variation across the layers when the LFP theta across all layers was lost.

3) The argument being put forward is that “cancellations and rhythm loss occurs due to interactions between OLM cells' synaptic and return currents and excitatory inputs”. An additional factor that could account for the loss of rhythmicity is the loss of theta frequency activity of the interneurons. Was the theta-frequency firing of interneurons validated for each simulation? The theta rhythmicity of inhibition should be reported.

As discussed above, due to our model structure and exploration, we would have an ongoing theta rhythm due to the imposed theta-frequency EPSC inputs to the inhibitory populations. As such, the mentioned additional factor for the loss of theta rhythmicity (in LFP) being due to the loss of theta frequency activity of the interneurons is not possible. To show this aspect more clearly, we have shown the full extent (5 sec) of the raster plots used in the examples shown in Figure 9 (and where we have also added schematics) so that the theta rhythmicity can be clearly seen. Also, as noted in the Methods, raster plots for all of the inhibitory network simulations (with their theta rhythmicity) are available on osf.io/vw3jh.

4) Similarly, assessing the relative power in theta vs other frequencies would be informative. For example, the present analysis does not distinguish between irregular LFP, and a reduced theta power (eg. as exemplified in the three panels of Fig9B). Calculating the ratio of theta to a different band (e.g. delta) could be informative.

As discussed above, due to our model structure, we would have ongoing theta rhythms due to the imposed theta-frequency EPSCs. Loss of theta occurs due to ‘cancellations with synaptic and return currents’ in the LFP generation stage. As such, considering other frequency bands in a direct fashion via power ratios would not be appropriate in our model structure.

5) On the whole, the writing is very dense. It would be helpful if schematics are included in diagrams wherever possible. These should show interneuron activity (high/low rate and rhythmicity) and the dominant currents. For example, in Fig 9, the 6 panels would be much easier to understand if there was a visual schematic for each.

We thank the reviewer for his/her suggestion. Schematics have been added to Figure 9, as well as Figure 10 to make them easier to understand.

Minor points: 1) The choice of abbreviation g_sb and g_bs is not evident. Perhaps change to g_ob/g_bo? While we agree that ‘ob’ and ‘bo’ are probably more appropriate subscripts to use, we prefer to keep ‘sb’ and ‘bs’ because of its former usage in Ferguson et al. (2015), and on which this work is built. We think that if we adjust it in the present manuscript, it would become confusing to readers of this paper and Ferguson et al. (2015).

10

2) The abbreviations PV and SOM need to be stated in first instance in the methods (line 112) Thank you, corrected in the manuscript

3) Line 117: It is not clear what is meant by peak alignments. Are these alignments to the phase of the theta oscillation? Yes, these alignments are to the phase of the LFP theta oscillation in experiment. We have expanded the relevant sentence to specifically state this. Specifically, as noted in Ferguson et al. 2015 (where figure references removed to avoid confusion): “EPSCs received by PV+ and SOM+ cells are quite precisely timed with respect to the peak of the LFP. However, to target specifically PV+ or SOM+ interneurons, our voltage clamp recordings were done separately on PV-tdTomato and SOM-tdTomato mice, respectively. Thus, to simulate the effect of simultaneously recorded PV+ and SOM+ EPSC recordings, we developed an algorithm to cut and shift the EPSCs. In this way, the two recordings exhibited EPSCs at the same frequency, and the EPSC peaks aligned. We will refer to these frequency-matched currents as EPSC_PV and EPSC_OLM.”

4) Line 188 state that spike rasters for BiC wre used to model excitatory input. What was the motivation for the selection of these specific BiC rasters? Please also specify c_sb. We did not have explicit spiking pyramidal cell models (point neurons with feedback etc.) in the model structure. Pyramidal cells are loosely phase locked to theta and fire sparsely during theta rhythms (Huh et al. 2016). Because of this, we considered the case where BiC inputs and the PYR cells inputs are temporarily correlated and thus chose the same spike time traces for BiC and pyramidal cell inputs to the multi-compartment PYR cell generating the LFP. This correlation would maximize any potential cancelation between pyramidal and BiC inputs in the basal dendrites. This was done, to explore whether excitatory-inhibitory input cancellations in the basal dendrites can be responsible for the lower LFP power recorded in that region, a reasonable assumption based on the experimental LFPs. We have added the c_sb value of 0.21.

5) Lines 209 - 218. This discussion of results is out of place in the methods section and difficult to understand. We have now removed this from the Methods. After some consideration, we decided to simply exclude it as it is perhaps an excessive detail for the given point.

6) Lines 242, repetition of 142-143 This repetition is now removed.

7) Figures 1, 2A, 10, 11 should use labeled sub-panels. It is difficult to follow with references to “top”, “bottom” This has been done.

8) Figure 2A: It is confusing that the dark is above the light for all the rasters, but that it is inverted for the LFP trace. This has been adjusted.

9) The text from lines 414-442 is very difficult to follow. The “four regions” in the 2A colour plot are not evident. Addition of schematics in the figure to accompany the text would be helpful. We have made the four regions more obvious on the Figure 2A colour plot (dotted lines separating the regions which are also labelled) and adjusted the accompanying text referring the additional parts and schematics, which have now also all been labelled.

11

10) Lines 461-470 set out various predictions for the full model, but these are not referred to again. It would be helpful for the reader if each of these predictions are addressed explicitly and revisited. We now explicitly refer back to these observations and predictions of Figure 2 A,B in the later part of the Results.

11) What was the upper band for theta frequency used The upper bound of LFP theta frequency is 6 Hz, as imposed by the EPSCs (see line 497).

12) Figure 5 legend should be changed to “...parameter sets using different LOWER frequency bounds” Thank you. This is now done.

13) Lines 558-563: I do not understand the argument being put forward about the OLM cell LFP component. Please rephrase We were essentially comparing direct (OLM-PYR) and indirect (OLM-BiC-PYR) pathway contributions by OLM cells, where we had earlier referred to direct and indirect pathways. We have rephrased and expanded these lines to make it clear.

14) Line 585-586: “It is clear from the magnitude of the signal power that it is indeed mainly due to the component from PV cells”. The reasoning in this sentence seems awry. The signal is due to the PV cells, because that is how the network has been set up. But this is not seen from the magnitude of the signal power. (line 565-6). What we meant was PV cells versus OLM cells contributing to the power. We have expanded this sentence to clarify.

15) Legend to Fig 7: Please specify that the plots show peak power at electrode of maximal LFP theta power. We have expanded the legend to Figure 7 to specify this.

16) Figure 7A: Inset is too small Inset has now been made larger.

17) Line 584: Why are you considering g_bs=0 for mimicking OLM optogenetic silencing? The bistratified synapses would still be functional in this situation Broadly speaking, in this scenario our objective is to assess the contribution of OLM cells and thus we remove them from the network to see how the LFP will be affected by their absence. We refer to this set of simulations as the approximate equivalent to “optogenetic silencing” as equally, in the case of optogenetic silencing, one is interested in the effect of the absence of a given cell type to the network output. The computational approach to simulate cell silencing can be considered by removing the connections coming from the particular cell type in the network by setting those synaptic conductances to zero. We could’ve instead chosen to silence the cell units directly and maintain the synapses, this wouldn’t have made a difference in our results as the maintained synapses would have been dormant since there is no cell to generate presynaptic spiking and activate the synapse.

In Figure 7C, it theoretically does not matter that we set conductance from bistratified onto OLM (g_bs) to zero to mimic OLM cell optogenetic silencing because OLM cell to PYR cell connections are removed and thus OLM cells cannot affect the LFP characteristics regardless of how their activity is modulated by g_bs. As can be seen in Figure 7C, the whole column for all g_bs values (including zero) has the same output (i.e., colour is the same) exactly because OLM projections on the PYR are removed.

12

18) Lines 586: “Consider that for the parameter regime g_sb=0 ...” is this referring to the intact model in 7A, or the PV only network of 7C? Please specify. We are referring to the intact model, Figure 7A. We have expanded this sentence to specify this.

19) For the text accompanying Fig 9, it would be helpful to state explicitly what you are trying to show in this figure. Why were the particular examples selected? We observed that the LFP output (with PV or OLM cells removed) could be approximately grouped into certain categories based on their laminar LFP profiles. Figure 9 shows representative examples of the characterized LFP profile. We have reorganized the accompanying text in the revised manuscript to make this clear.

20) Lines 639-644: Figure 9 does not reveal that that the contribution of OLM cells is more dependent on g_bs due to the colour scaling. This is only apparent in Fig 10, middle, left. These lines seem to be saying the same thing three times. Please make this more concise. We agree that Figure 9 does not allow one to visualize this dependence -- Figure 7 and 10 were intended. As noted in the Figure 10 legend, the color scaling was adjusted to allow a comparison and visualization to be apparent. We have rephrased to be more concise.

21) Line 657-666: These are discussion points. These points have now been moved to the Discussion (‘Theta rhythms and summary of results’ section).
