# Supplementary material for: Deciphering the Contribution of Oriens-Lacunosum/Moleculare (OLM) Cells to Intrinsic θ Rhythms Using Biophysical Local Field Potential (LFP) Models
Source: eNeuro. 2018 Sep 4;5(4):ENEURO.0146-18.2018. doi: 10.1523/ENEURO.0146-18.2018 (PMC6140113; doi:10.1523/ENEURO.0146-18.2018)
Supplement: Supplementary file 2 — Supplementary Code. Download supplement 2, GZ file [file sup_enu-eN-NWR-0146-18-s02.gz › LFP_microcircuit-master/gsb=0.00_gbs=0.00_csb=0.21_LFP_and_somatic_potential_one_electrode.pdf]

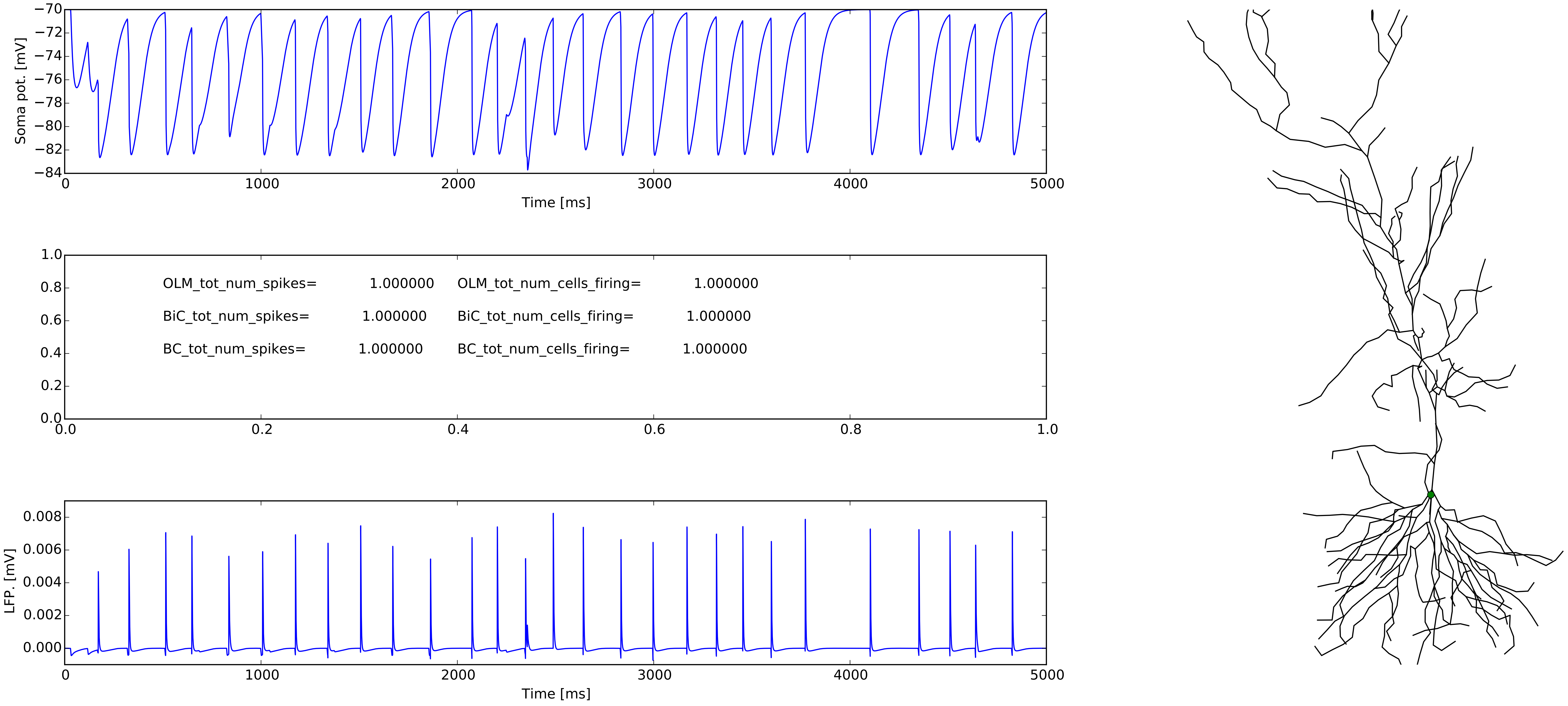
Soma pot. [mV]
Time [ms]
OLM_tot_num_spikes= 1.000000
OLM_tot_num_cells_firing= 1.000000
BiC_tot_num_spikes= 1.000000
BiC_tot_num_cells_firing= 1.000000
BC_tot_num_spikes= 1.000000
BC_tot_num_cells_firing= 1.000000
LFP. [mV]
Time [ms]
